# Solving Chemical Absorption Equilibria using Free Energy and Quantum Chemistry Calculations: Methodology, Limitations, and New Open-Source Software

**DOI:** 10.1021/acs.jctc.3c00144

**Published:** 2023-04-20

**Authors:** H. Mert Polat, Frédérick de Meyer, Céline Houriez, Othonas A. Moultos, Thijs J. H. Vlugt

**Affiliations:** † Engineering Thermodynamics, Process & Energy Department, Faculty of Mechanical Engineering, Delft University of Technology, Leeghwaterstraat 39, Delft 2628CB, The Netherlands; ‡ CCUS R&D Program, Gas & Low Carbon Entity, OneTech, TotalEnergies S.E., 92078 Paris, France; ¶ Mines Paris, PSL University, Center for Thermodynamics of Processes (CTP), 77300 Fontainebleau, France

## Abstract

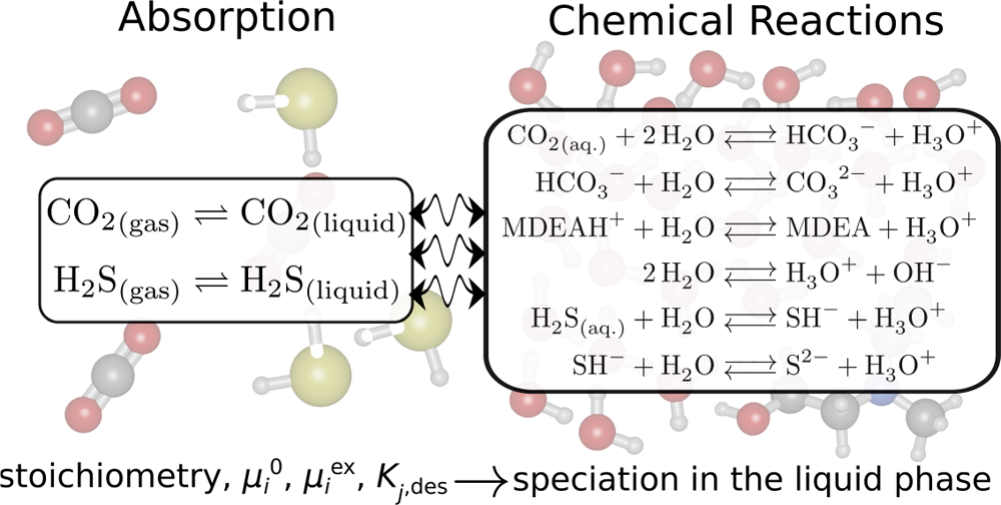

We developed an open-source chemical reaction equilibrium solver in Python (CASpy, https://github.com/omoultosEthTuDelft/CASpy) to compute the concentration of species in any reactive liquid-phase absorption system. We derived an expression for a mole fraction-based equilibrium constant as a function of excess chemical potential, standard ideal gas chemical potential, temperature, and volume. As a case study, we computed the CO_2_ absorption isotherm and speciation in a 23 wt % *N*-methyldiethanolamine (MDEA)/water solution at 313.15 K, and compared the results with available data from the literature. The results show that the computed CO_2_ isotherms and speciations are in excellent agreement with experimental data, demonstrating the accuracy and the precision of our solver. The binary absorptions of CO_2_ and H_2_S in 50 wt % MDEA/water solutions at 323.15 K were computed and compared with available data from the literature. The computed CO_2_ isotherms showed good agreement with other modeling studies from the literature while the computed H_2_S isotherms did not agree well with experimental data. The experimental equilibrium constants used as an input were not adjusted for H_2_S/CO_2_/MDEA/water systems and need to be adjusted for this system. Using free energy calculations with two different force fields (GAFF and OPLS-AA) and quantum chemistry calculations, we computed the equilibrium constant (*K*) of the protonated MDEA dissociation reaction. Despite the good agreement of the OPLS-AA force field (ln[*K*] = −24.91) with the experiments (ln[*K*] = −23.04), the computed CO_2_ pressures were significantly underestimated. We systematically investigated the limitations of computing CO_2_ absorption isotherms using free energy and quantum chemistry calculations and showed that the computed values of μ_*i*_^ex^ are very sensitive to the point charges used in the simulations, which limits the predictive power of this method.

## 1 Introduction

Accurately solving chemical reaction equilibria is a challenging numerical problem with significant importance to many industrial processes^1,2^ such as steam reforming of methane and formic acid,^3^ and acid gas (CO_2_ and H_2_S) capture from flue gas or natural gas streams.^4,5^ By using correct thermodynamic and numerical methods, chemical information can be obtained for conditions that are difficult to measure experimentally, such as high temperatures and pressures, or experiments with dangerous materials.^6^ Solving chemical reaction equilibrium allows us to have access to the speciation, i.e., the concentration of each species at equilibrium, which often requires tedious experimental spectroscopic measurements.^7−9^ It is very challenging to solve the chemical reaction equilibria of systems without reliable experimental data.^10^ In this case, free energy calculations using molecular simulations and quantum chemistry calculations are very advantageous. Two thermodynamic properties are crucial to solve reaction equilibria accurately using free energy and quantum chemistry calculations: (1) the excess chemical potential μ_*i*_^ex^ and (2) the standard state ideal gas chemical potential μ_*i*_^0^. μ_*i*_^ex^ describes the affinity of species *i* with the surrounding medium, and the affinity of reactants and reaction products to the solvent influences chemical equilibria.^2^ Since the activity coefficient of species *i* (γ_*i*_) also describes the affinity of species *i* with the surrounding medium, μ_*i*_^ex^ is related to γ_*i*_^11−13^ as
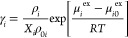
1where ρ_*i*_ is the number density of species *i*, *X*_*i*_ is the mole fraction of species *i*, ρ_0*i*_ is the reference number density of the pure solvent (in the same units as ρ_*i*_), and μ_*i*0_^ex^ is the excess chemical potential of species *i* in pure solvent. Using free energy calculations, the values of μ_*i*_^ex^ can be computed.^4,5,14,15^ The μ_*i*_^0^ of reactants and reaction products also influences chemical equilibria since μ_*i*_^0^ is related to the molar Gibbs free energy of the pure substance *i*.^16^ Quantum chemistry calculations can be used to compute μ_*i*_^0^.^4,5,17^ A methodology for computing chemical reaction equilibrium constants using free energy and quantum chemistry calculations have been already established in the literature.^4,17−20^

The chemical reaction equilibrium in a solvent can be solved using Reaction Ensemble Monte Carlo (RxMC) simulations.^21−25^ In RxMC simulations, reactants and products of a reaction can be interconverted through insertions and deletions of molecules to compute the speciation at chemical equilibrium.^14,23,26^ Smith and Qi^18^ described a novel algorithm called Reaction Ensemble Molecular Dynamics (REMD) to predict chemical reaction equilibria in MD simulations. In REMD simulations, the Gibbs free energy is iteratively minimized by changing the composition in the simulation box. Smith and Qi^18^ investigated N_2_/O_2_/NO and N_2_/H_2_/NH_3_ systems using the REMD algorithm. The compositions obtained using the REMD algorithm agreed with experiments and simulations from the literature.

Noroozi et al.^19^ developed a methodology for the calculation of chemical reaction equilibrium constants in the liquid phase using molecular simulations and quantum chemistry calculations and investigated CO_2_/monoethanolamine (MEA)/water systems. In this study, it was reported that predicting the concentration of minor species such as the bicarbonate ion HCO_3_^–^ or free CO_2_ in the solution, and the CO_2_ isotherms in aqueous MEA solutions are very challenging. Noroozi et al.^4^ also computed the equilibrium constants for the reactions of 7 different primary/secondary alkanolamines and CO_2_, and the carbamated alkanolamine dissociation reaction for these 7 primary/secondary alkanolamines. Although the equilibrium constants and the concentration of minor species at equilibrium computed by Noroozi et al.^4^ did not always agree with the values from the literature, the CO_2_ absorption isotherms showed a reasonable agreement with experimental isotherms from the literature. In another study, Noroozi et al.^17^ computed the values of p*K*_a_ of protonated alkanolamine dissociation reactions for 29 different alkanolamine species using three different methods: (1) quantum chemistry calculations at three different levels of theory (Hartree–Fock (HF)),^27^ second order Møller–Plesset perturbation theory (MP2),^28^ and Becke’s three parameter hybrid exchange functional with Lee–Yang–Parr correlation functional (B3LYP),^29,30^ (2) the SMD continuum solvent method, and (3) the AM1-BCC point charge assignment method. These authors^17^ showed that none of the investigated methods can predict the values of p*K*_a_ that consistently agree with experiments. Noroozi et al.^20^ determined a new force field for the hydronium ion (H_3_O^+^) by fitting the computed p*K*_a_ to the experimental p*K*_a_ of a well-known system (CO_2_/MEA/water). Using this force field for H_3_O^+^, the values of p*K*_a_ for 77 different alkanolamines were predicted. The authors showed that the predicted values of p*K*_a_ have an average absolute deviation of 0.72 in units of p*K*_a_ (i.e., an absolute deviation of 1.66 in units of ln[*K*] since ln[*K*] = ln[10] × p*K*_a_) from the experimental values in the literature. The average absolute deviation of 1.66 ln[*K*] units corresponds to a change of ca. 5.25 times in units of *K*, and this is too high to accurately compute the speciation in systems that are very sensitive to the value of the protonated amine dissociation reaction equilibrium constant. Therefore, it is important to investigate the limitations of the method.

**Figure 1:**
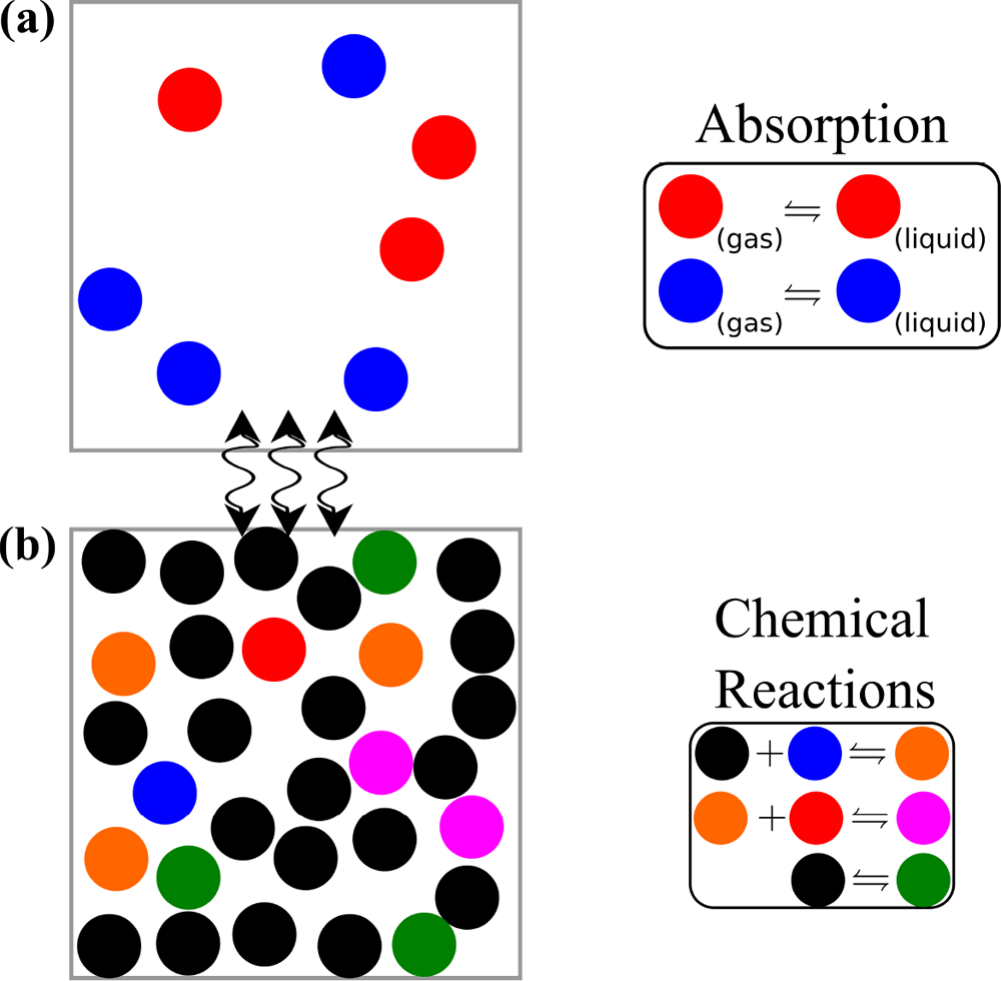
Schematic representation of our chemical reaction equilibrium solver. (a) Different species in the gas phase are absorbed by (b) the liquid phase where the absorbed species undergo chemical reactions. Given the stoichiometry of the reactions in the liquid phase, the values of μ_*i*_^ex^ and μ_*i*_^0^ of species, and/or the desired equilibrium constants of the reactions in the liquid phase, CASpy computes the speciation in the liquid phase at equilibrium. The partial pressures of the species in the gas phase at equilibrium can also be computed using the concentrations and excess chemical potentials of these species in the liquid phase.

In this study, we develop a chemical reaction equilibrium solver in Python called CASpy and use it to solve binary (and single-component) CO_2_ and H_2_S absorption isotherms in aqueous methyldiethanolamine (MDEA) solutions (see the Supporting Information for source code for CASpy version 0.1.6). We study the absorption of CO_2_ and H_2_S in aqueous MDEA solutions because it is relevant to biogas upgrading^31^ and acid gas (CO_2_ and H_2_S) removal from natural gas.^32−36^ Molecular simulation is a natural choice for this application as simulations allow studies without the difficulty of working with H_2_S (due to safety and environmental concerns),^37^ and eliminate the low accuracy of experiments at low partial pressures of acid gases.^38,39^ For this purpose, we first derive an expression for the equilibrium constant as a function of μ_*i*_^ex^ and μ_*i*_^0^ of species *i*, temperature, and volume using a mole fraction-based equilibrium constant and develop software to solve chemical reaction equilibria in combination with absorption. A schematic representation of the scheme of our chemical reaction equilibrium solver is shown in Figure 1. The species in the gas phase absorb into the liquid phase where the chemical reactions occur. We assume that the volume of the liquid phase and μ_*i*_^ex^ of the species do not change with composition, and compute the speciation in the liquid phase at equilibrium using the reaction stoichiometry, the values of μ_*i*_^ex^ and μ_*i*_^0^, and/or the desired equilibrium constants. CASpy can be used for any reaction network in the liquid phase. We present the accuracy of the solver using two case studies: (1) CO_2_/MDEA/water and (2) H_2_S/CO_2_/MDEA/water. We showcase the accuracy and precision of the solver by comparing the computed CO_2_ isotherms in aqueous MDEA with experimental isotherms from the literature, and by comparing the speciation at equilibrium for the CO_2_/MDEA/water system with the respective experimental data. We also compared the binary absorption isotherms of H_2_S and CO_2_ in aqueous MDEA with the available data from the literature. To assess the sensitivity of the absorption isotherms, we also computed the equilibrium constant of the protonated MDEA dissociation reaction using two different force fields, the General Amber Force Field (GAFF)^40^ and the OPLS-AA force field.^41,42^ We use these equilibrium constants to compute CO_2_ isotherms in aqueous MDEA solutions and compare the computed isotherms with experimental data. We quantify the sensitivity of μ_*i*_^ex^ and the equilibrium constant for protonated MDEA dissociation reaction to the point charges of the species.

This manuscript is organized as follows: the thermodynamic framework, case studies, and simulation methods are discussed in the next section. In section 3, we present and discuss the results from modeling and simulations and compare our results with available literature data. In section 4, we discuss our conclusions regarding the modeling of reactive systems and limitations of using free energy and quantum chemistry calculations for modeling reactive systems.

## 2 Methods

### 2.1 Chemical Reaction Equilibrium Solver

The equilibrium of a chemical reaction occurs when the sum of chemical potentials of the reaction products times the stoichiometric coefficient of the reaction products is equal to that of the reactants at constant temperature and pressure. The equilibrium condition for a chemical reaction can be formulated as^2^
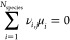
2where *N*_species_ is the number of species involved in reaction *j* (including the solvent and the solutes), ν_*i*,*j*_ is the stoichiometric coefficient of species *i* in reaction *j*, and μ_*i*_ is the chemical potential of species *i*. For the remainder of this study, we consider the stoichiometric coefficients of the reaction products positive, while the reactants have negative stoichiometric coefficients. The chemical potentials of solutes are calculated using an ideal gas reference frame:^14^
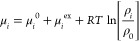
3where μ_*i*_^0^ is the standard state ideal gas chemical potential^4,14^ of species *i*, μ_*i*_^ex^ is the excess chemical potential of species *i*, *R* is the ideal gas constant, *T* is the absolute temperature, ρ_*i*_ is the number density of solute *i* in the solvent, and ρ_0_ is the reference number density of 1 molecule Å^–3^. Note that different reference states can be used to compute chemical potentials.^43^ In this study, we use ρ_0_ as reference state for chemical potentials to be consistent with our previous work.^14,15^ Other definitions for the chemical potential using different reference states can also be used to compute chemical reaction equilibria with the methodology described in this work.^44^ However, a conversion of reference states will be required. The chemical potential of the solvent (μ_s_) in a solution is computed with the ideal gas reference state using^2^
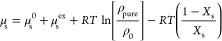
4where ρ_pure_ is the number density of the pure solvent and *X*_s_ is the mole fraction of the solvent *s* in the solution (*X*_s_ = *N*_s_/*N*_total_ where *N*_total_ is the sum of number of molecules of all species in the solution including the solvent). The term  in eq 4 originates from the Gibbs–Duhem equation at constant temperature and pressure.^45^

The equilibrium constant of reaction *j* (*K*_*j*_) can be defined using the mole fraction of each species in the solution as
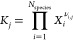
5where *X*_*i*_ is the mole fraction of species *i*. Using the equilibrium condition of eq 2, we derive the desired equilibrium condition of reaction *j* (*K*_*j*,des_) as a function of μ_*i*_^0^, μ_*i*_^ex^, *T*, and volume *V* as
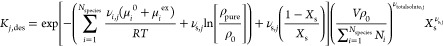
6where ν_s,*j*_ is the stoichiometric coefficient of the solvent in reaction *j* and ν_total solute,*j*_ is the sum of stoichiometric coefficients of the solutes (all species except for the solvent) in reaction *j*. This means that at chemical equilibrium, *K*_*j*_ = *K*_*j*,des_. A detailed derivation of eq 6 is provided in the Supporting Information. The required values of μ_*i*_^0^ and μ_*i*_^ex^ to calculate *K*_*j*,des_ can be computed using quantum chemistry calculations and molecular simulations, respectively. eq 6 implies that the mole fraction of the solvent *X*_s_ is constant. In CASpy, we solve the value of *X*_s_ iteratively. This means that a new *K*_*j*,des_ is computed based on the new mole fraction of the solvent after solving for the speciation of the system at equilibrium. This is performed until the difference between the new mole fraction of the solvent and the old mole fraction no longer changes. In practice, the difference in the speciation between solving the value of *X*_s_ iteratively and assuming a constant value of *X*_s_ is very small. Using the computed speciation in the liquid phase at equilibrium, the partial pressure of the gas species *i* (*P*_*i*_) can be computed using
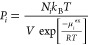
7where *N*_*i*_ is the number of molecules of species *i* in the liquid phase, *V* is the volume of the liquid phase which is constant, and *k*_B_ is the Boltzmann constant. Alternatively, using CASpy, the total pressure and the composition of the gas phase can be imposed, and the solver computes the speciation in the liquid phase (and the absorbed amount). Note that when the total pressure and the composition of the gas phase are imposed, the mass balance equations for the gases should not be used since there is mass transfer from the infinite gas phase to the liquid phase. Also, the addition of nonreactive gases (such as N_2_ or CH_4_ in aqueous MDEA solutions) will not influence the outcome of our model.

In this study, we use the “least_squares” function as implemented in the Scipy^46^ library in Python to solve for the speciation (the number of molecules or concentration of each species in the solution) of the liquid phase at equilibrium. The “least_squares” is a function for solving nonlinear equations with the least-squares method. This requires an objective function to be defined. The objective function is computed by summing the squares of the values of individual equations (residuals). CASpy runs until the value of each residual is lower than 10^–10^ to ensure that the global minimum of the objective function is obtained. CASpy can be used to compute the speciation in any reactive liquid phase. The objective function is constructed using the following residuals:
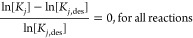
8
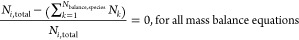
9
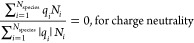
10where *N*_balance,species_ is the number of species included in the mass balance equation, *q*_*i*_ is the net charge of species *i*, and *N*_species_ is the total number of species in the solution. Note that including charge neutrality (eq 10) to our set of equations is necessary because we use molecule-based balance equations (eq 9) and the net charge of each molecule is fixed. With element-based balance equations (i.e., carbon, oxygen, hydrogen, and nitrogen balances), the charge neutrality would not be required as long as the net charge of each element is fixed. However, the stoichiometry of each molecule and ion should be known with the element-based balance equations. To generalize our solver, we used molecule-based balance equations with the addition of the charge neutrality. An example input file and the detailed explanation of the input file are provided in the Supporting Information.

### 2.2 Case Study

As a case study for CASpy, we investigate the binary CO_2_ and H_2_S absorption from an ideal gas phase to aqueous MDEA. In the CO_2_/MDEA/water system, we have four reactions:^36^
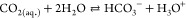
R1
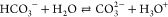
R2
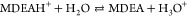
R3
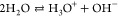
R4By combining reaction R1 and the reverse reaction R3 (−R3), we can obtain the reaction between CO_2_, MDEA, and water . There are 8 species in the CO_2_/MDEA/water system, including the reaction products. These species are the free CO_2_, water (solvent), HCO_3_^–^, H_3_O^+^, CO_3_^2–^, MDEA, MDEAH^+^, and OH^–^. The equilibrium constants for each of these reactions can be computed using eq 5, and the equilibrium follows from *K*_*j*_ = *K*_*j*,des_. In this work, the values of *K*_*j*,des_ for the reactions R1, R2, and R4 are computed using the correlations provided by Plakia et al.^36^ since these reactions are present in systems where CO_2_ is absorbed by an aqueous solution of any primary, secondary, or tertiary alkanolamine. We computed the desired equilibrium constant of the MDEAH^+^ dissociation reaction (*K*_R3,des_) either by using the correlation provided by Plakia et al.,^36^ or by performing MC simulations and quantum chemistry calculations. The correlations to compute mole fraction-based equilibrium constants reported by Plakia et al.^36^ are listed in Table S30 of the Supporting Information. Note that the logarithm of an equilibrium constant (p*K*_a_) can be converted to natural logarithm of the equilibrium constant  using . For this system, four additional equations must be satisfied at equilibrium: the MDEA balance, CO_2_ balance, water balance, and charge neutrality. The MDEA balance equals:
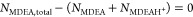
11The water balance equals:
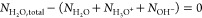
12The CO_2_ balance equals:
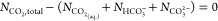
13Finally, charge neutrality of the system is formulated as
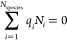
14Thus, for the CO_2_/MDEA/water system, we have 8 different species and 8 equations to satisfy.

With the addition of H_2_S, two additional reactions are added to the reaction network, which are^47^
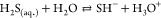
R5
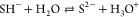
R6By combining reaction R5 and the reverse reaction R3 (−R3), we obtain the reaction between H_2_S and MDEA (H_2_S + MDEA *⇌* MDEAH^+^ + SH^–^). While the combination of reactions R5 and R3 is kinetically favorable,^48^ our reaction network (reactions R1–R6) is thermodynamically consistent, meaning that we can compute the equilibrium constant of the combined reaction of reactions R5 and R3 using the equilibrium constants of reactions R5 and R3. Note that this is also valid for the combined reaction of reactions R1 and R3. The addition of the component H_2_S to the CO_2_/MDEA/water system leads to an additional equation for the H_2_S balance. The H_2_S balance equals:
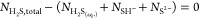
15With the addition of H_2_S to the CO_2_/MDEA/water system, we have 3 additional species in the solution. These species are the free H_2_S, bisulfide ion SH^–^, and sulfide ion S^2–^. We also have 3 more equations to solve which are *K*_R5_ = *K*_R5,des_, *K*_R6_ = *K*_R6,des_, and the H_2_S balance. In summary, in the H_2_S/CO_2_/MDEA/water system, we have 11 different species and 11 equations to satisfy.

The objective function for CO_2_/MDEA/water systems is defined as an array of eq 8 and the following residuals:
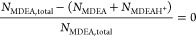
16
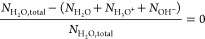
17
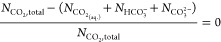
18
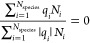
19The objective function for H_2_S/CO_2_/MDEA/water systems is defined as an array of eq 8, eqs 16–19, and an additional residual for H_2_S balance in the system:
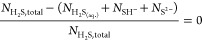
20Each residual in the objective functions should be equal to 0 at equilibrium. Note that each residual in the objective functions (eqs 8–10) is normalized to make sure that the residuals are of similar magnitudes. We also need to scale the initial guess for the speciation to unity to ensure that the numerical solver will deal with variables of similar magnitudes.^49^ Otherwise, there will be a ca. 12 orders of magnitude difference between the concentration of the most scarce species in the solution (H_3_O^+^) and the concentration of the most abundant one (water). This would make it challenging to numerically find a solution at equilibrium. For this purpose, at the start of our calculations, the variable array is divided by itself (element-wise) and kept in the memory (scaling factors). While computing the residuals, we scale the variable array back by multiplying the solution with the scaling factors stored in the memory. In our calculations, we use 10^–15^ as both termination tolerance for individual variables (number of species of each species) and for the residuals. The tolerances are sufficiently low since we use normalized residuals (eqs 8–10).

### 2.3 Monte Carlo Simulations

We perform MC simulations in the *NPT* ensemble to compute the values of μ_*i*_^ex^. To this purpose, we use Brick-CFCMC,^14,15,24^ an open source state-of-the-art MC simulation software for computing phase- and reaction equilibria. Brick-CFCMC uses efficient Continuous Fractional Component Monte Carlo (CFCMC)^14,15,23,24,50,51^ methods for molecule insertions and deletions. CFCMC uses the so-called “fractional” molecule groups to insert or delete molecules from the simulation box. A “fractional” molecule group can contain multiple molecules and/or ions as long as it is charge neutral. The interactions between the “fractional” molecule group and the surrounding molecules are scaled using a parameter called λ. At λ = 0, the “fractional” molecule group has no interactions with the surrounding molecules while at λ = 1, the “fractional” molecule group has full interactions with the surrounding molecules.^23,25,50,51^ There are two different methods implemented in Brick-CFCMC to compute μ_*i*_^ex^. The details of these methods are explained in the Supporting Information. In this study, we compute μ_*i*_^ex^ using thermodynamic integration.^15,26,52^ For thermodynamic integration, we compute the ensemble average of the derivative of the potential energy with respect to the interaction scaling factor ⟨∂*U*/∂λ⟩ for 50 equidistant and fixed values of λ. We also compute the value of the term ⟨∂*U*/∂λ⟩ at λ = 10^–6^ and λ = 1–10^–6^ to increase the accuracy of the thermodynamic integration.

The TIP3P^53^ force field was used to model water in this study. We used this force field because the μ_*i*_^ex^ of water computed using the TIP3P agrees with experimental μ_*i*_^ex^ much better than the μ_*i*_^ex^ computed using the TIP4P or the TIP5P force fields.^54^ In Brick-CFCMC, the value of ⟨∂*U*/∂λ⟩ can only be computed for a charge-neutral group of “fractional” molecules. For this purpose, we included a rigid HCO_3_^–^ ion to the fractional group of either MDEAH^+^ or H_3_O^+^ (Table S1 of the Supporting Information). The choice of counterion does not matter because the value of μ_*i*_^ex^ of the counterion cancels out when we compute *K*_R3,des_ using eq 6. For flexible MDEA and MDEAH^+^, we used either the General Amber Force Field (GAFF)^40^ with RESP fitted point charges or the OPLS-AA force field^41,42^ with 1.14*CM1A point charges.^55^ For H_3_O^+^ ions, we used the force field developed by Noroozi et al.^20^ Details of the MC simulations including all force field parameters can be found in the Supporting Information (Figures S1–S4 and Tables S2–S28 of the Supporting Information).

### 2.4 Quantum Chemistry Calculations

In this study, we perform quantum chemistry calculations using the Gaussian09 software^56^ to compute the values of μ_*i*_^0^ for the MDEAH^+^ ion, the MDEA molecule, the H_3_O^+^ ion, and water. As the MDEAH^+^ ion and the MDEA molecule have many different conformers (molecules with different spatial arrangements), we first conducted a conformer search for these molecules.^57^ We optimized the structure of 5 different conformers for both MDEAH^+^ and MDEA with the Gaussian-4 (G4) composite method^58^ and chose the conformers with the minimum free energy. The molecular partition function computed in these calculations were used to compute μ_*i*_^0^. Details on computing μ_*i*_^0^ using quantum chemistry calculations are explained in the Supporting Information. We also compute the electrostatic potential energy grid of the conformers at the minimum free energy using the Merz–Kollman scheme^59^ at the Hartree–Fock (HF)^27^ level of theory with a 6-31G* basis set. The computed electrostatic potential energy grids are used in a two-step Restrained Electrostatic Potential Surface (RESP) fitting with the Antechamber package^60^ to compute the point charges of these molecules for the GAFF.^40^

## 3 Results and Discussion

### 3.1 Absorption of CO_2_ in Aqueous MDEA Solutions

**Figure 2:**
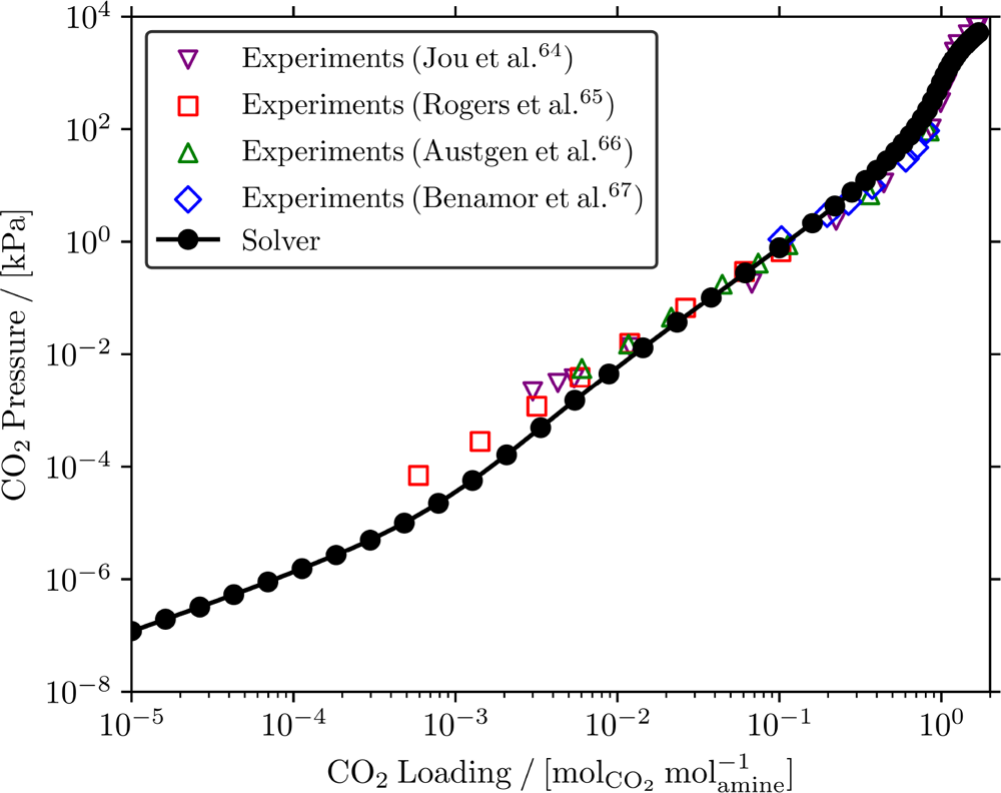
Comparison of the calculated and experimental CO_2_ isotherms^65−68^ in 23 wt % MDEA/water solutions at 313.15 K. Note that the experimental values of *K*_*j*,des_ provided by Plakia et al.^36^ were used for all reactions in the CO_2_/MDEA/water system (R1–R4) in the calculations with the solver (Table S30 of the Supporting Information).

As a first case study, we investigate CO_2_ absorption in aqueous MDEA. Based on the definition of mole fraction-based reaction equilibrium constant (eq 5), we assume an ideal solution where the activity coefficients of all species are constant. The activity coefficients of species can be computed from μ_*i*_^ex^ in the solution and μ_*i*_^ex^ in pure solvent^11−13^ using eq 1. In principle, the activity coefficients of species can be computed using an activity coefficient model or iteratively.^61,62^ The latter means that a new set of μ_*i*_^ex^ can be calculated based on the speciation computed using the values of μ_*i*_^ex^ at infinite dilution, and this can be performed until the differences between the old values and new values of μ_*i*_^ex^ no longer change. However, it was previously shown that the speciation obtained by the ideal solution assumption and the nonideal case are very similar for CO_2_ absorption in aqueous alkanolamine solutions.^4^ We implemented the specific ion interaction theory (SIT)^63,64^ with our chemical reaction equilibrium solver to test if the ideal solution assumption differs from the nonideal case. The results show that the differences are indeed very small. Therefore, the results presented in this study are obtained with the ideal solution assumption. In these calculations, we use the experimental values of *K*_*j*,des_ provided by Plakia et al.^36^ for all 4 reactions in the CO_2_/MDEA/water system (R1–R4) at 313.15 K (see Table S30 of the Supporting Information for the correlations). To compute the partial pressure of CO_2_ using the concentration of free CO_2_ in the liquid phase at equilibrium (eq 7), we computed the value of μ_*i*_^ex^ for CO_2_ in water at 313.15 K and 1 bar. The values of μ_*i*_^ex^ for CO_2_ in water as a function of temperature are listed in Table S31 of the Supporting Information. To validate that CASpy yields the correct solutions at equilibrium, we investigate the sum of the square of the residuals ( where *N*_obj_ is the number of residuals in the objective function and *V*_*i*_ is the value of residual *i*) as a function of the CO_2_ loading in the solution. Our results show that the sum of the squared residuals is 0 within machine precision for all CO_2_ loadings. This means that the solutions computed by CASpy are at chemical equilibrium. Figure 2 shows the computed CO_2_ pressure as a function of the CO_2_ loading along with the experimental CO_2_ isotherms from the literature^65−68^ in 23 wt % MDEA/water solutions at 313.15 K.

**Figure 3:**
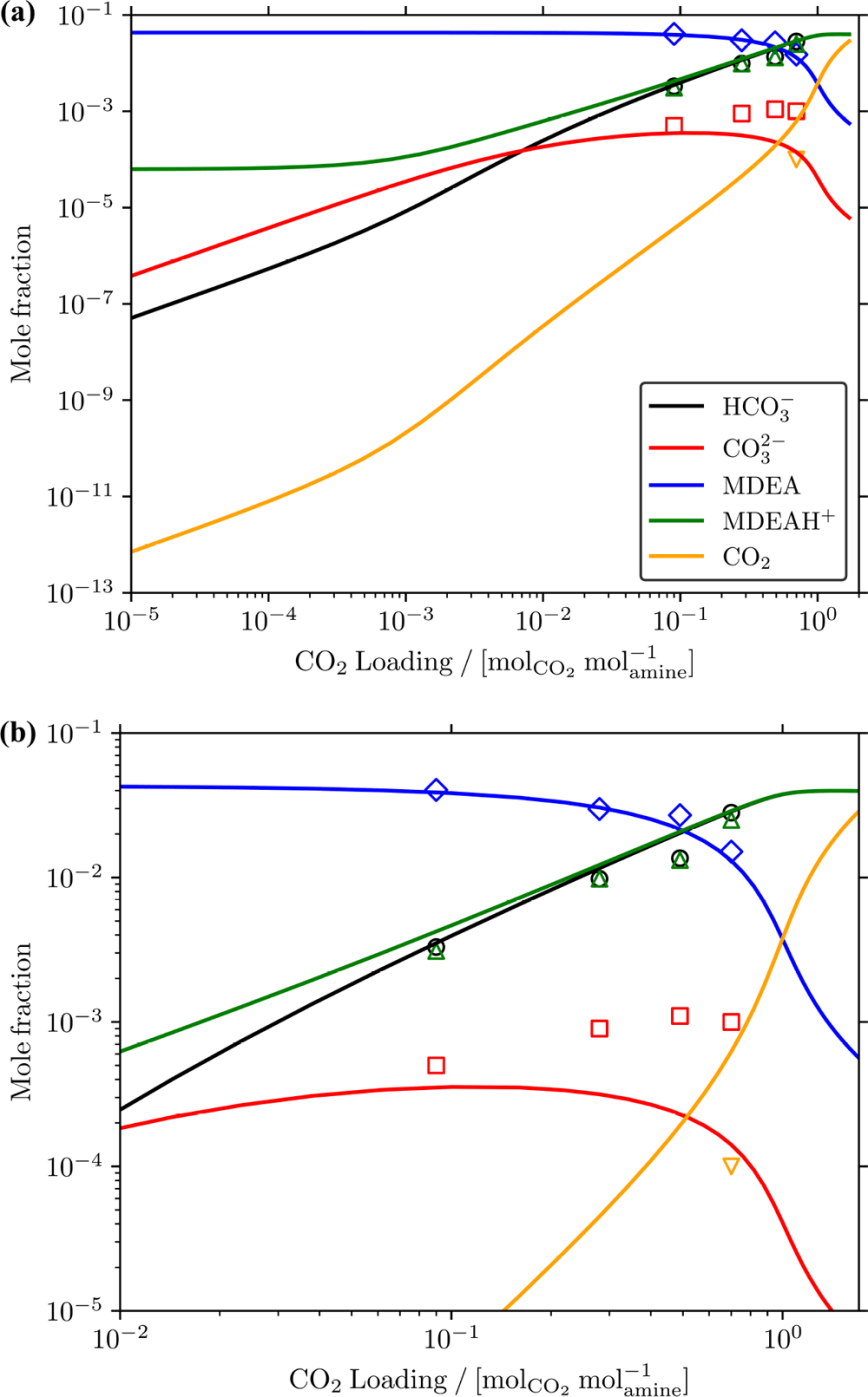
Comparison of the calculated and experimental^7^ speciations of CO_2_ loaded 23 wt % MDEA/water solutions at 313.15 K for a CO_2_ loading range between (a)  and (b) . Open symbols represent the experimental speciation reported by Jakobsen et al.;^7^ □: CO_3_^2–^, ▽: CO_2_, ◇: MDEA, △: MDEAH^+^, ○: HCO_3_^–^. Note that the experimental values of *K*_*j*,des_ provided by Plakia et al.^36^ were used for all reactions in the CO_2_/MDEA/water system (R1–R4) in our calculations (Table S30 of the Supporting Information). The color coding in (b) follows that of (a).

It is clearly shown that the computed CO_2_ pressures are in excellent agreement with the experiments from the literature. Figure 2 also shows that the computed CO_2_ pressures are slightly lower than the experimental pressures at low loadings (<). This may be because the experiments at low pressures of CO_2_ are less accurate than the experiments at higher CO_2_ pressures.^38,39^ Motivated by this excellent agreement, we also compare experimental^7^ and calculated speciations in CO_2_ loaded 23 wt % MDEA/water solutions at 313.15 K. Figure 3 shows the experimental speciation from the literature^7^ and the calculated speciation as a function of CO_2_ loading in 23 wt % MDEA/water at 313.15 K.

The comparison of the speciations shows that the calculated concentrations of MDEA, MDEAH^+^, and HCO_3_^–^ agree well with the experimental measurements for all CO_2_ loadings. However, this is not the case for the concentration of free CO_2_ and the carbonate ion CO_3_^2–^. For CO_3_^2–^, the calculated concentration at the lowest loading agrees well with the experimental measurements, while the CO_3_^2–^ concentrations are underpredicted for higher loadings. Jakobsen et al.^7^ state that the measured CO_3_^2–^ concentrations are most likely overestimated at high CO_2_ loadings. This was shown by the excess negative charge that Jakobsen et al.^7^ reported. An excess negative charge means that the net charge of the system is not zero but negative, so the concentration of CO_3_^2–^ is overestimated. For the free CO_2_ concentration, the chemical reaction equilibrium solver slightly overpredicts the only experimental measurement that Jakobsen et al.^7^ reported. However, these authors state that the measured free CO_2_ concentration may be underestimated due to the chemical exchange between the species at equilibrium complicating the integration of the NMR spectra.

### 3.2 Binary Absorption of CO_2_ and H_2_S in Aqueous MDEA Solutions

**Figure 4:**
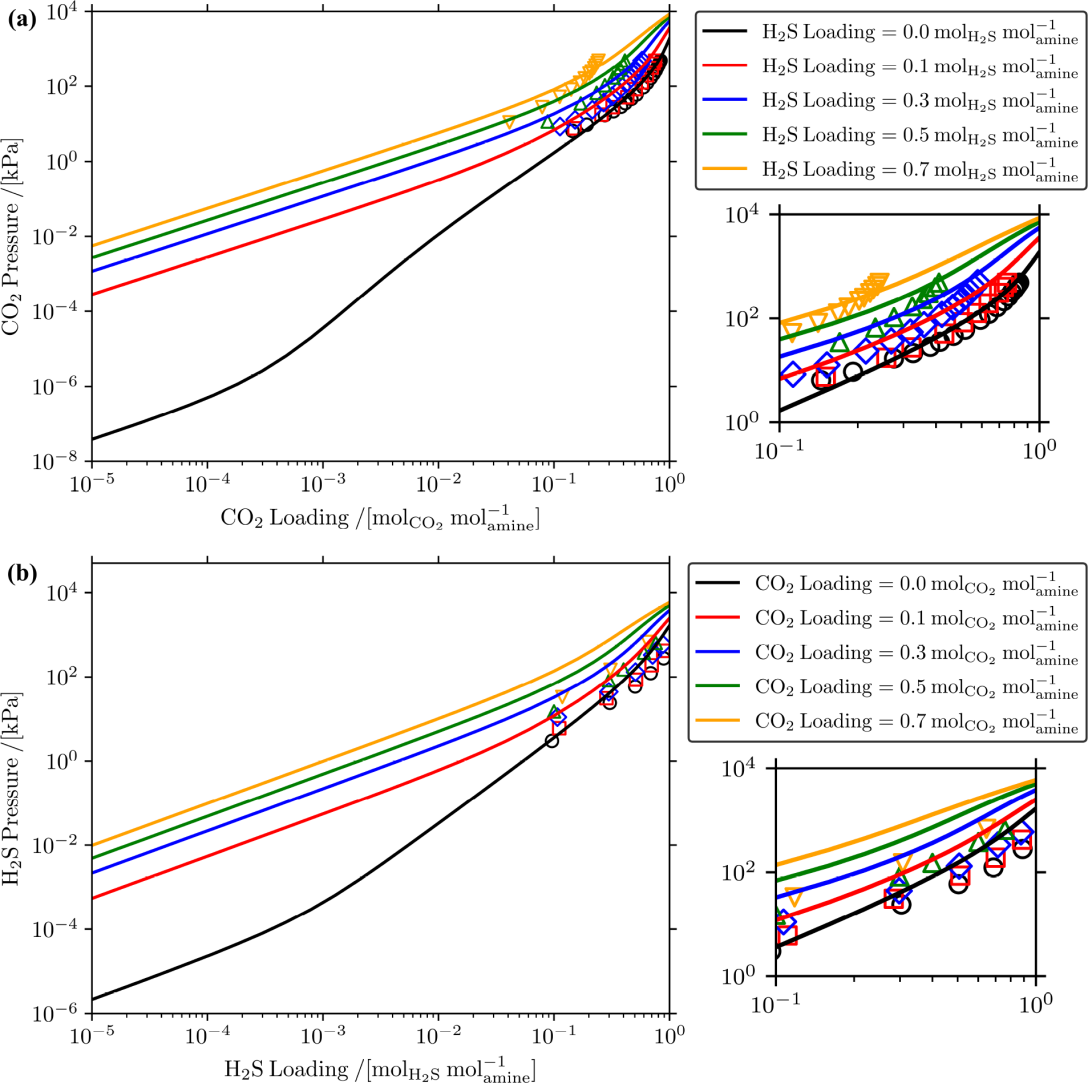
Absorption isotherms of (a) CO_2_ and (b) H_2_S in 50 wt % MDEA/water solutions at 313.15 K and fixed H_2_S and CO_2_ loadings, respectively. Open symbols represent modeling and experimental results from Dicko et al.^69^ for (a) and (b), respectively. The color coding for the open symbols follows that of the solid lines. The figures below the legends show the CO_2_ (H_2_S) pressures in kPa for a CO_2_ (H_2_S) loading range between 0.1 to 1 . Note that experimental values of *K*_*j*,des_ provided by Plakia et al.^36^ were used for all reactions in H_2_S/CO_2_/MDEA/water system (R1–R6) for the calculations in the solver (Table S30 of the Supporting Information).

As a second case study, we investigate the binary absorption of CO_2_ and H_2_S in aqueous MDEA. To this purpose, we computed the values of μ_*i*_^ex^ for H_2_S in water as a function of temperature at 1 bar. The values of μ_*i*_^ex^ for H_2_S in water as a function of temperature are listed in Table S31 of the Supporting Information. We computed the CO_2_ and H_2_S isotherms in 50 wt % MDEA/water solutions at 323.15 K and fixed H_2_S and CO_2_ loadings, respectively. Note that all values of the residuals in these calculations were 0 within machine precision, thus, the solutions correspond to chemical equilibrium. Dicko et al.^69^ performed a modeling study on the CO_2_ and H_2_S isotherms in aqueous MDEA solutions for fixed H_2_S and CO_2_ loadings, respectively. These authors also reported experimental H_2_S isotherms in aqueous MDEA solutions at fixed CO_2_ loadings. Figure 4 shows the comparison between the calculated absorption isotherm of CO_2_ (and H_2_S) in 50 wt % MDEA/water solution at 313.15 K and fixed H_2_S (and CO_2_) loading, and modeling (and experimental) data from Dicko et al.^69^Figure 4(a) shows that with increasing loading of H_2_S, CO_2_ pressure also increases. The same behavior can be seen for H_2_S pressures as a function of CO_2_ loading in Figure 4(b). This effect is more prominent at low acid gas loadings. Figure 4 also shows that the calculated CO_2_ isotherms at fixed H_2_S loading are in agreement with the modeling results from Dicko et al.^69^ at higher CO_2_ loadings. At lower loadings (total acid gas loading <1 mol_acidgas_ mol_amine_^–1^), the results from the two models deviate significantly. For H_2_S isotherms, Figure 4(b) shows that the calculated H_2_S isotherms in 50 wt % MDEA/water solution at fixed CO_2_ loadings do not agree well with the experimental results by Dicko et al.^69^ The sequential binary absorption of CO_2_ first and H_2_S second approach by Dicko et al.^69^ may be the reason for the difference between our H_2_S isotherms and experimental results. It is important to note that there may be CO_2_ evaporating in the second part of the measurement due to the competitive absorption with H_2_S. We modified our solver so we can quantify this effect. Details of this correction are explained in the Supporting Information. The CO_2_ loading and H_2_S pressure were computed as a function of H_2_S loading using the modified solver. Figure S5 shows the CO_2_ loading as a function of H_2_S loading during H_2_S absorption. Our results show that CO_2_ indeed evaporates from the solution to the gas phase during H_2_S absorption.^69^ The amount of evaporated CO_2_ is the lowest at the lowest CO_2_ loading. For the initial CO_2_ loading of 0.093 , the decrease in the CO_2_ loading is 0.6–26.9% of the initial amount, while for the highest CO_2_ loading, the decrease is 9.5–44.7% of the initial CO_2_ loading. Figure S6 of the Supporting Information shows the H_2_S isotherms for the fixed CO_2_ loading assumption, by the effect of evaporating CO_2_, and the experimental results from Dicko et al.^69^Figure S6 shows that the H_2_S pressure decreases for fixed H_2_S loadings when we account for the evaporation of CO_2_. This is because there is less CO_2_ in the solution for H_2_S to compete with. The decrease in H_2_S pressure is 0.6–5.9% for the initial CO_2_ loading of 0.093 , while the decrease in H_2_S pressure is between 21.6–37.6% for the highest initial CO_2_ loading (i.e., 0.706 ).

Even after accounting for the effect of CO_2_ evaporation during H_2_S loading, the computed H_2_S isotherms still do not agree well with the experimental results from Dicko et al.^69^ For example, Dicko et al.^69^ measured the H_2_S pressure as 680 kPa for a CO_2_ loading of 0.706  and a H_2_S loading of 0.645  in 50 wt % MDEA/water solution at 323.15 K, while the computed H_2_S pressure is 1887 kPa at the same conditions. Even when there is no CO_2_ in the solution (CO_2_ loading = 0 ), the calculated H_2_S isotherm does not agree with the experimental results from Dicko et al.^69^ except for the data point at the lowest H_2_S loading. For a H_2_S loading of 0.884 , the calculated pressure of H_2_S is 996 kPa, while the experimental H_2_S pressure is 278 kPa. This may be because of two reasons; (1) we use experimental values of *K*_*j*,des_ for all reactions R1–R6) reported by Plakia et al.^36^ However, these parameters were not fitted to binary absorption of CO_2_ and H_2_S in aqueous MDEA. Thus, experimental values of *K*_*j*,des_ may be less accurate for the conditions we are investigating. (2) We use the μ_*i*_^ex^ of infinitely diluted H_2_S in water at 323.15 K to compute H_2_S pressure using eq 7. This means that we assume that the μ_*i*_^ex^ of CO_2_ and H_2_S do not change with the increasing concentration of CO_2_, H_2_S, and different ions (see R1–R6). We tested this assumption by computing the μ_*i*_^ex^ of CO_2_ for different CO_2_ loadings in 23 wt % MDEA/water solutions at 313.15 K using the speciations reported in Figure 3. Figure S7 of the Supporting Information shows the computed data. Our data show that the difference between the μ_*i*_^ex^ of CO_2_ at the highest CO_2_ loading  and the lowest CO_2_ loading  is well within the chemical accuracy (1 kcal mol^–1^ = 4.18 kJ mol^–1^).^70^

### 3.3 Sensitivity and Limitations of the Method

We tested the sensitivity of computed CO_2_ pressures in aqueous MDEA solutions to the computed values μ_*i*_^0^ and μ_*i*_^ex^ by computing *K*_*j*,des_ of the MDEAH^+^ dissociation reaction R3 using eq 6. We used either the GAFF^40^ with point charges fitted with RESP or the OPLS-AA force field^41,42^ with 1.14*CM1A point charges^55^ for MDEAH^+^ and MDEA. For water, we used the TIP3P force field,^53^ while we used the optimized force field by Noroozi et al.^20^ for the H_3_O^+^ ions. Using CFCMC simulations and thermodynamic integration,^15^ we computed the μ_*i*_^ex^ of MDEAH^+^ (HCO_3_^–^ as the counterion), MDEA, H_3_O^+^ (HCO_3_^–^ as the counterion), and water. We also computed the μ_*i*_^0^ of MDEAH^+^, MDEA, H_3_O^+^, and water using quantum chemistry calculations. The values of μ_*i*_^ex^ and μ_*i*_^0^ of MDEAH^+^, MDEA, H_3_O^+^, and water are listed and compared with the available data from the literature in Tables S32 and S33 of the Supporting Information. We also listed the values of μ_*i*_^0^ and the atomizations energies (*D*_0,*i*_) computed with different quantum chemistry composite methods in Table S34 of the Supporting Information. The computed values of μ_*i*_^0^ show that different quantum chemistry composite methods result in very similar values of μ_*i*_^0^ as the standard deviations are between 1.5–2.6 *k*_B_*T*. Note that every 1 *k*_B_*T* unit change in values of μ_*i*_^0^ corresponds to a change of ca. 1 in terms of  (eq 6). Also, Table S32 of the Supporting Information shows that the calculated values of μ_*i*_^0^ agree with the values computed using the JANAF tables^71,72^ within 6–8 *k*_B_*T* for charge neutral molecules (water and CO_2_), while the difference between the values of μ_*i*_^0^ computed using quantum chemistry calculations and JANAF tables^71,72^ for ions (H_3_O^+^ and OH^–^) are between 5–17 *k*_B_*T*. Since the standard deviation between the values of μ_*i*_^0^ computed using different quantum chemistry composite methods in Gaussian09^56^ is low and the G4 method is one of the most accurate methods,^58^ we use the G4 method to compute the values of μ_*i*_^0^ for the remainder of this study. We compared the values of μ_*i*_^ex^ computed using MC simulations with available experimental data from the literature and values of μ_*i*_^ex^ computed from Henry constant of species in water (Table S33 of the Supporting Information). Our results show that the computed values of μ_*i*_^ex^ agree with the available data from the literature within the chemical accuracy (1 kcal mol^–1^ = 4.18 kJ mol^–1^).^70^ Using the computed values of μ_*i*_^ex^ and μ_*i*_^0^, we computed the equilibrium constant of the MDEAH^+^ dissociation reaction *K*_R3,des_ at 313.15 K (eq 6). The natural logarithms of the computed values of *K*_R3,des_ for different force fields are listed in Table 1.Our results show that the computed *K*_R3,des_ using the OPLS-AA force field^41,42^ with point charges derived from 1.14*CM1A^55^ agrees well with the experimental^36^ value, while the value computed using GAFF^40^ with RESP fitted point charges differs from the experiments. Noroozi et al.^17^ computed the p*K*_a_ of protonated amine dissociation reactions for 29 different alkanolamine species at 298.15 K and 1 bar. To make the results of Noroozi et al.^17^ comparable with our study, we convert the values of p*K*_a_ these authors report to the units of  Noroozi et al.^17^ compared the values of p*K*_a_ computed using GAFF with RESP fitted point charges, SMD continuum solvent simulations, and GAFF with the semiempirical AM1-BCC charge model with experimental values of p*K*_a_ from the literature. For the RESP fitting, these authors computed the electrostatic potential of the species at 3 different levels of theory using quantum chemical calculations. The authors showed that although some calculated values of p*K*_a_ agree with the experimental data within 1 p*K*_a_ unit, none of the investigated methods is consistently successful in accurately predicting p*K*_a_ of protonated amine dissociation reactions. For example, Noroozi et al.^17^ computed the value of  of protonated MDEA dissociation reaction between 28.1 and 31.5, while the experimental value from the literature is 23.8^73^ at 298.15 K. Noroozi et al.^17^ also showed that the deviations in computed values of p*K*_a_ are quite large for some alkanolamines. For example, these authors computed the value of  of protonated tris(hydroxymethyl)aminomethane (THMAM) dissociation reaction between 9.9 and 20.7, while the experimental value from the literature is 22.7 at 298.15 K.

**Table 1:** Natural Logarithms of the Computed Values of *K*_R3,des_ (Reaction R3) for the GAFF and OPLS-AA Force Field and Natural Logarithm of the Experimental Value^36^ of *K*_R3,des_ at 313.15 K

	ln[KR3,des]	Source
GAFF	–34.80	This work
OPLS-AA	–24.91	This work
Experimental	–23.04	Plakia et al.^36^

**Figure 5:**
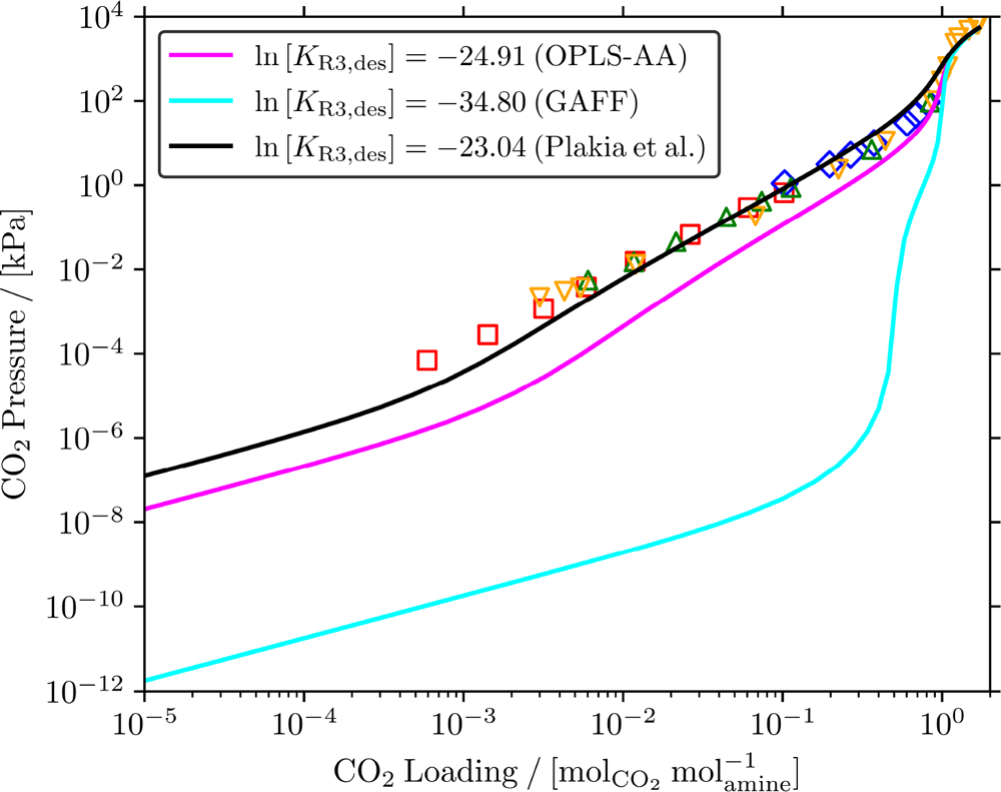
Comparison of experimental CO_2_ isotherms^65−68^ and the calculated CO_2_ isotherms obtained using *K*_R3,des_ from the OPLS-AA force field,^41,42^ the GAFF,^40^ and the experimental correlation from Plakia et al.^36^ in 23 wt % MDEA/water solution at 313.15 K. Note that experimental values of *K*_*j*,des_ provided by Plakia et al.^36^ were used for reactions R1, R2, and R4 in the CO_2_/MDEA/water system, while for reaction R3, we used *K*_R3,des_ from the OPLS-AA force field, GAFF, or the experimental correlation from Plakia et al.^36^ The solid lines represent the CO_2_ isotherms computed with CASpy while the empty symbols represent CO_2_ isotherms from the literature.^65−68^ The color codes of the empty points (experiments) follow those in Figure 2.

To test the sensitivity of CO_2_ isotherm in an aqueous MDEA solution to the value of *K*_R3,des_, we computed the CO_2_ isotherm in 23 wt % MDEA/water solution at 313.15 K using the values of μ_*i*_^0^ computed from quantum chemistry calculations and the values of μ_*i*_^ex^ computed using thermodynamic integration. Figure 5 shows the CO_2_ isotherms computed using *K*_R3,des_ from OPLS-AA force field, GAFF, and the experimental correlation from Plakia et al.,^36^ and experimental CO_2_ isotherms from the literature^65−68^ as a function of CO_2_ loading. As clearly shown in Figure 5, when *K*_R3,des_ computed with GAFF  is used, the computed CO_2_ pressures are significantly underestimated at low CO_2_ loadings (<1 ), while at high loadings (>1 ), the computed CO_2_ pressures agree well with the experimental isotherms. The computed CO_2_ pressures were underestimated at low CO_2_ loadings because lower values of *K*_R3,des_ mean that reaction R3 is dominated by the species on the left side of the reaction (MDEAH^+^ and H_2_O) (Eq. 5). This means that the CO_2_ dissociation reaction (reaction R1) proceeds toward the right side of the reaction more freely, so more CO_2_ is absorbed by the solution (in the form of HCO_3_^–^, and consequently CO_3_^2–^) at low CO_2_ loadings. This results in the underestimation of the CO_2_ isotherm at low loadings of CO_2_ computed using *K*_R3,des_ from GAFF. When *K*_R3,des_ computed with the OPLS-AA force field is used, the agreement between the computed and experimental CO_2_ isotherms is much better than GAFF but still differs from the experimental isotherms. At the lowest CO_2_ loading , the CO_2_ pressure computed using *K*_R3,des_ from the experimental correlation reported by Plakia et al.^36^ is ca. 6 times higher than the CO_2_ pressure computed using *K*_R3,des_ from the OPLS-AA force field. At a higher CO_2_ loading , the CO_2_ pressure computed using *K*_R3,des_ from the experimental correlation reported by Plakia et al.^36^ is ca. 16 times higher than the CO_2_ pressure computed using *K*_R3,des_ from the OPLS-AA force field. The isotherms computed using the GAFF and OPLS-AA force field agree well with the experimental CO_2_ isotherms at high CO_2_ loadings. This is because the limit of chemical CO_2_ absorption in aqueous MDEA solutions is the CO_2_ loading of 1  (due to the one-to-one stoichiometry between CO_2_ and MDEA in reactions R1–R4). At loadings higher than 1 , we only have physical absorption of CO_2_ in the solution. This can also be seen with the changing slope of the CO_2_ isotherms at CO_2_ loadings higher than 1 . The only parameter affecting the amount of physically absorbed CO_2_ in our model is the μ_*i*_^ex^ of CO_2_. This shows that we predict the μ_*i*_^ex^ of CO_2_ in water correctly, therefore, all the isotherms agree with the experimental CO_2_ isotherms at high CO_2_ loadings. All in all, Figure 5 shows that the computed CO_2_ isotherms are sensitive to the changes in the equilibrium constant of reaction R3 (*K*_R3,des_). Even with a reasonable prediction of the value of *K*_R3,des_ (ln[*K*_R3,des_](OPLS-AA) = −24.91 vs ln[*K*_R3,des_](Plakia et al.) = −23.04) from quantum chemistry calculations and MC simulations, the CO_2_ isotherms computed are quite different.

To investigate absorption at low pressures, we derived an expression for the Henry constant of CO_2_ in aqueous MDEA solutions. Details of derivation of the expression for the Henry constant of CO_2_ are shown in the Supporting Information. The Henry constants computed using the expression we derived and computed using the slope of the CO_2_ isotherm (the one with *K*_R3,des_ from Plakia et al.^36^) show an excellent agreement since the Henry constant computed using the expression we derived is 0.0162  and the Henry constant computed using the slope of the CO_2_ isotherm is 0.0149 . We also validated the expression for the Henry constant of CO_2_ in aqueous MDEA solutions using the speciation obtained from CASpy. Table S29 of the Supporting Information shows the excellent agreement between the speciation computed using the Henry constant expression we derived and the speciation computed numerically with our solver. This means that the expression derived for the Henry constant of CO_2_ in aqueous MDEA solutions can be used to accurately compute absorption at low pressures.

**Figure 6:**
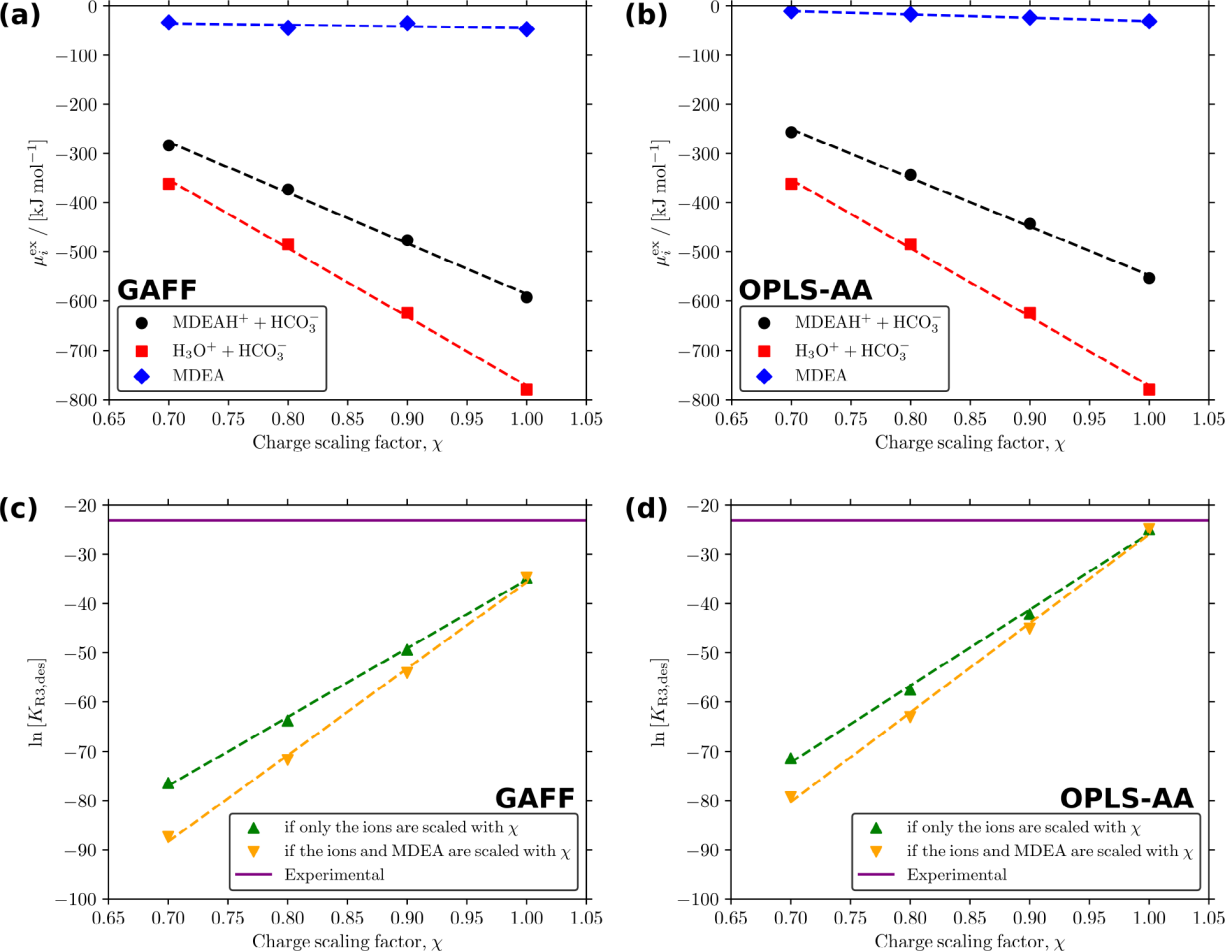
(a,b) Values of μ_*i*_^ex^ of the species in reaction R3, and (c,d) values of  for (a,c) the GAFF with RESP fitted point charges and (b,d) the OPLS-AA force field with 1.14*CM1A point charges as a function of the point charge scaling factor χ at 313.15 K. The dashed lines in all subfigures represent the linear regression fits to the values of μ_*i*_^ex^ (the fit parameters are tabulated in Tables S35 and S36 of the Supporting Information).

To test the sensitivity of computed values of *K*_R3,des_ to point charges, we computed the μ_*i*_^ex^ of MDEAH^+^ (and HCO_3_^–^ as counterion), H_3_O^+^ (and HCO_3_^–^ as counterion), and MDEA with point charge scaling factors (χ) of 0.9, 0.8, and 0.7 at 313.15 K. For example, χ = 0.9 means that all the point charges in the molecule were multiplied by 0.9. Figure 6 shows the computed values of μ_*i*_^ex^ and the computed values of  for GAFF with RESP fitted point charges and OPLS-AA force field with 1.14*CM1A point charges as a function of χ at 313.15 K. The parameters of the linear regression fits in Figure 6 are tabulated in Tables S35 and S36 of the Supporting Information.

The results show that for both force fields, the values of μ_*i*_^ex^ are very sensitive to the point charges. For GAFF with RESP fitted point charges, the value of μ_*i*_^ex^ for MDEAH^+^+HCO_3_^–^ increases by 95.63 kJ mol^–1^ (36.73 *k*_B_*T*) when χ = 0.9 is used instead of the unscaled point charges. The change in the value of μ_*i*_^ex^ for H_3_O^+^+HCO_3_^–^ is even more sensitive to the point charges (also seen in Table S35 of the Supporting Information with a lower slope). The value of μ_*i*_^ex^ for H_3_O^+^+HCO_3_^–^ increases by 155.53 kJ mol^–1^ (59.74 *k*_B_*T*) when χ = 0.9 is used instead of χ = 1.0. Our results also show that  is very sensitive to the changes in the point charges. For the OPLS-AA force field with 1.14*CM1A point charges, the computed value of  changes from −24.91 to −42.19 if only the point charges of the ions in reaction R3 are scaled, and to −45.15 if the point charges of MDEA are scaled as well. All in all, Figure 6 shows that we need force fields with very accurate point charges to be able to accurately compute CO_2_ isotherms in aqueous alkanolamines. Polarizable force fields are usually more accurate than classical force fields^74−81^ because the ability to accurately quantify electrostatic interactions is essential (Figure 6). However, polarizable force fields are not implemented widely in the software packages and are computationally more expensive than classical force fields.^82,83^

## 4 Conclusions

We derived an expression for a mole fraction-based equilibrium constant as a function of μ_*i*_^ex^, μ_*i*_^0^, and *T*, and developed an open-source chemical reaction equilibrium solver in Python called CASpy for absorption of gases to reactive solutions, assuming that the values of μ_*i*_^ex^ and the liquid phase volume *V* are constant. CASpy can be used to compute the concentrations of the species in any reactive liquid phase, for example, aqueous alkanolamine solutions for CO_2_ and H_2_S capture, and CO_2_ capture in an aqueous solution for the electrochemical conversion of CO_2_. We first validated that CASpy yields the correct numerical solution at chemical equilibrium. Our results showed that the computed solutions are at chemical equilibrium since the sum of all residuals was 0 within machine precision. We computed CO_2_ isotherms in 23 wt % MDEA/water solutions at 313.15 K using experimental equilibrium constants from the literature^36^ for all reactions R1–R4) and compared the computed isotherms with experimental isotherms from the literature. The results are in excellent agreement with the experiments. We compared the computed speciation in the CO_2_/MDEA/water system with the experimental speciation from the literature,^7^ showing an excellent agreement. For low pressures, we derived and validated an analytic expression for the Henry constant of CO_2_ in aqueous MDEA solutions. We computed binary CO_2_ and H_2_S absorption isotherms in 50 wt % MDEA/water solutions at 323.15 K using experimental equilibrium constants from the literature^36^ for all reactions R1–R6. The computed CO_2_ isotherms in the H_2_S/CO_2_/MDEA/water system show a good agreement with another modeling study from the literature;^69^ however, the computed H_2_S isotherm in the H_2_S/CO_2_/MDEA/water system did not agree well with experimental isotherms by Dicko et al.^69^ As these authors first performed CO_2_ absorption and then H_2_S absorption in a 50 wt % MDEA/water solution, and did not account for the CO_2_ evaporating in the H_2_S absorption part of the experiment, we also estimated the amount of evaporated CO_2_ by making some modifications to our solver. The H_2_S isotherm computed considering the effect of evaporating CO_2_ agreed better with the experimental results^69^ than the H_2_S isotherm computed without considering the effect of evaporating CO_2_. However, agreement with the experimental results from Dicko et al.^69^ is lacking. This implies that the experimental equilibrium constants^36^ from the literature were not suitable for H_2_S/CO_2_/MDEA/water systems and the equilibrium constants of the reactions in H_2_S/CO_2_/MDEA/water systems need to be refitted. We tested the sensitivity of the computed CO_2_ isotherms in aqueous MDEA solutions to the computed values of μ_*i*_^ex^ and μ_*i*_^0^ by computing these values for the MDEAH^+^ dissociation reaction R3 in water at 313.15 K and 1 bar using MC simulations and quantum chemistry calculations. Two different force fields for MDEAH^+^ and MDEA were used in the MC simulations (GAFF^40^ and OPLS-AA^41,42^). Using the computed values of μ_*i*_^ex^ and μ_*i*_^0^ and eq 6, we computed the value of *K*_R3,des_ at 313.15 K. The value of *K*_R3,des_ computed using the OPLS-AA force field *K*_R3,des_ (ln[*K*_R3,des_](OPLS-AA) = −24.91) showed a good agreement with the experimental value ( (Plakia et al.^36^) = −23.04) from the literature while the value of *K*_R3,des_ computed using the GAFF  differed from the experimental value. We computed the CO_2_ isotherms in 23 wt % MDEA/water solutions at 313.15 K using the experimental equilibrium constants from the literature^36^ for reactions R1, R2, and R4, while we used either *K*_R3,des_ computed using the GAFF or the OPLS-AA force field. Results showed that the computed CO_2_ isotherms are in an excellent agreement with the experimental isotherms at high CO_2_ loadings (>1 ). However, the difference between the computed CO_2_ isotherms and the experimental isotherms is quite large for lower CO_2_ loadings (<1 ). Even with a good agreement between the value of *K*_R3,des_ computed using the OPLS-AA force field and the experimental value of *K*_R3,des_ from the literature, the computed CO_2_ pressures were 6 and 12 times lower than the experimental isotherms at  and , respectively. This shows that the CO_2_ isotherm in aqueous MDEA solutions is very sensitive to the value of *K*_R3,des_. Furthermore, we computed the values of μ_*i*_^ex^ and *K*_R3,des_ for the GAFF and OPLS-AA force field and for charge scaling factors χ of 0.9, 0.8, and 0.7. Our results showed that even with a 10% change in the point charges, the changes in the values of μ_*i*_^ex^ and *K*_R3,des_ were very large. The value of μ_*i*_^ex^ for MDEAH^+^+HCO_3_^–^ has increased by ca. 37 *k*_B_*T* from χ = 1.0 to χ = 0.9, while the value of μ_*i*_^ex^ for H_3_O^+^+HCO_3_^–^ has increased by ca. 60 *k*_B_*T*. The value of  computed using the GAFF has decreased from −34.80 to −49.42 when charges are scaled by χ = 0.9, while the value of  computed using the OPLS-AA force field decreased from −24.91 to −42.19. Our results show that force fields with accurate point charges are required to be able to solve chemical reaction equilibrium accurately. Further research must be conducted to develop accurate point charge assignment methods.

## Data Availability

The chemical reaction equilibrium solver CASpy can be downloaded from https://github.com/omoultosEthTuDelft/CASpy and https://pypi.org/project/CASpy-ReactionEquilibria/.

## Supporting Information Available

The source code for CASpy (version 0.1.6, 22/03/2023) (ZIP)
Derivation of an expression for mole fraction-based reaction equilibrium constants (section S1), detailed explanation of the input file for chemical reaction equilibrium solver (section S2), details of computing μ_*i*_^0^ with quantum chemistry calculations and the JANAF tables (sections S3.1 and S3.2), details of computing μ_*i*_^ex^ using Brick-CFCMC (section S3.3), simulation details including all force field parameters (section S4), details of accounting for CO_2_ evaporation in sequential absorption of CO_2_ and next H_2_S (section S5), derivation of an expression for the Henry constant of CO_2_ in aqueous MDEA solutions (section S6) (PDF)
