# Supplementary material for: Solving Chemical Absorption Equilibria using Free Energy and Quantum Chemistry Calculations: Methodology, Limitations, and New Open-Source Software
Source: J Chem Theory Comput. 2023 Apr 20;19(9):2616–29. doi: 10.1021/acs.jctc.3c00144 (PMC10173452; doi:10.1021/acs.jctc.3c00144)
Supplement: Supplementary file 2 — ct3c00144_si_002.pdf [file ct3c00144_si_002.pdf]

Supporting Information for: Solving Chemical Absorption Equilibria using Free Energy and Quantum Chemistry Calculations: Methodology, Limitations, and New Open-Source Software

H. Mert Polat,[†] Frédérick de Meyer,[‡,¶] Céline Houriez,[¶] Othonas A. Moultos,[†] and Thijs J.H. Vlugt[*,†]

†*Engineering Thermodynamics, Process & Energy Department, Faculty of Mechanical Engineering, Delft University of Technology, Leeghwaterstraat 39, Delft 2628CB, The Netherlands*

‡*CCUS R&D Program, Gas & Low Carbon Entity, OneTech, TotalEnergies S.E., 92078 Paris, France*

¶*Mines Paris, PSL University, Center for Thermodynamics of Processes (CTP), 77300 Fontainebleau, France*

E-mail: t.j.h.vlugt@tudelft.nl

The following items are presented in this Supporting Information:

- Derivation of an expression for mole fraction-based reaction equilibrium constants (section S1),
- Detailed explanation of the input file for chemical reaction equilibrium solver (section S2),
- Details of computing μ_i^0 with quantum chemistry calculations and the JANAF tables (sections S3.1 and S3.2),
- Details of computing μ_i^{ex} using Brick-CFCMC (section S3.3),
- Simulation details including all force field parameters (section S4),
- Details of accounting for CO_2 evaporation in sequential absorption of CO_2 and next H_2S (section S5),
- Derivation of an expression for the Henry constant of CO_2 in aqueous MDEA solutions (section S6).

S1 Derivation of an Expression for Mole Fraction-based Reaction Equilibrium Constants

Our aim is to derive an expression for the reaction equilibrium constant K as a function of the standard ideal gas chemical potential of species i (μ_i^0), the excess chemical potential of species i (μ_i^{ex}), and the absolute temperature T. Using the equilibrium condition and the definitions of chemical potential for solutes and the solvent (Eqs. 3 and 4 of the main text), we derive an expression for desired equilibrium constant. The equilibrium condition of reaction j can be expressed as[1]:

$$\sum_{i=1}^{N_{\text{species}}} \nu_{i,j}\mu_i = 0 \tag{S1}$$

where N_{species} is the number of species involved in reaction j, μ_i is the chemical potential of species i, and $\nu_{i,j}$ is the stoichiometric coefficient of species i in reaction j. In our methodology, we assign positive stoichiometric coefficients to the reaction products while signed stoichiometric coefficients are assigned to the reactants. The chemical potential of solutes are calculated using[2]:

$$\mu_i = \mu_i^0 + \mu_i^{\text{ex}} + RT\ln\left[\frac{\rho_i}{\rho_0}\right] \tag{S2}$$

where ρ_i is the number density of the solute i in the solvent, ρ_0 is the reference number density of 1 molecule Å^{-3}, μ_i^0 is the standard state ideal gas chemical potential[2,3] of species i, μ_i^{ex} is the excess chemical potential of species i, R is the ideal gas constant, and T is the absolute temperature. The chemical potential of the solvent is computed with ideal gas reference state using[1]:

$$\mu_{\text{s}} = \mu_{\text{s}}^0 + \mu_{\text{s}}^{\text{ex}} + RT\ln\left[\frac{\rho_{\text{pure}}}{\rho_0}\right] - RT\left(\frac{1-X_{\text{s}}}{X_{\text{s}}}\right) \tag{S3}$$

where ρ_{pure} is the number density of the pure solvent and X_{s} is the mole fraction of the solvent s in the solution ($X_{\text{s}} = N_{\text{s}}/N_{\text{total}}$, N_{total} is the sum of number of molecules of all species in the solution including the solvent). As an example, let us assume a reaction A + S $\rightleftharpoons$ B + C where S is the solvent. Using Eq. (S1), Eq. (S2), and Eq. (S3), we can write[1]:

$$\begin{aligned} \nu_{\text{A},j}\mu_{\text{A}}^{0} + \nu_{\text{A},j}\mu_{\text{A}}^{\text{ex}} + \nu_{\text{A},j}RT\ln\left[\frac{\rho_{\text{A}}}{\rho_0}\right] \\ +\nu_{\text{B},j}\mu_{\text{B}}^{0} + \nu_{\text{B},j}\mu_{\text{B}}^{\text{ex}} + \nu_{\text{B},j}RT\ln\left[\frac{\rho_{\text{B}}}{\rho_0}\right] \\ +\nu_{\text{C},j}\mu_{\text{C}}^{0} + \nu_{\text{C},j}\mu_{\text{C}}^{\text{ex}} + \nu_{\text{C},j}RT\ln\left[\frac{\rho_{\text{C}}}{\rho_0}\right] \\ +\nu_{\text{S},j}\mu_{\text{S}}^{0} + \nu_{\text{S},j}\mu_{\text{S}}^{\text{ex}} + \nu_{\text{S},j}RT\ln\left[\frac{\rho_{\text{pure}}}{\rho_0}\right] - \nu_{\text{S},j}RT\left(\frac{1-X_{\text{S}}}{X_{\text{S}}}\right) = 0 \end{aligned} \tag{S4}$$

For an arbitrary chemical reaction, this can be rewritten as:

$$\begin{aligned} \exp\left[-\left(\sum_{i=1}^{N_{\text{species}}}\frac{\nu_{i,j}(\mu_i^0+\mu_i^{\text{ex}})}{RT} + \nu_{\text{s},j}\ln\left[\frac{\rho_{\text{pure}}}{\rho_0}\right]\right)\right](V\rho_0)^{\nu_{\text{total,solute},j}} = \\ \exp\left[-\nu_{\text{s},j}\left(\frac{1-X_{\text{s}}}{X_{\text{s}}}\right)\right]\prod_{i=1}^{N_{\text{solute}}} N_i^{\nu_{i,j}} \end{aligned} \tag{S5}$$

where N_{solute} is the number of solutes (excluding the solvent) in the reaction, N_i is the number molecules of the species i, $\nu_{\text{s},j}$ are the stoichiometric coefficient of the solvent in reaction j, and $\nu_{\text{total,solute},j}$ is the sum of the stoichiometric coefficients of all solutes in reaction j ($\sum_{i=1}^{N_{\text{solute}}}\nu_{i,j}$). We define the equilibrium constant of reaction j as:

$$K_j' = \exp\left[-\nu_{\text{S},j}\left(\frac{1-X_{\text{S}}}{X_{\text{S}}}\right)\right]\prod_{i=1}^{N_{\text{solute}}} N_i^{\nu_{i,j}} \tag{S6}$$

The left side of the Eq. (S5) can be used to compute the desired equilibrium constant K_{des}' as a function of μ_i^0, μ_i^{ex}, T, and V.

$$K'_{j,\mathrm{des}} = \exp\left[-\left(\sum_{i=1}^{N_\mathrm{species}} \frac{\nu_{i,j}(\mu_i^0 + \mu_i^\mathrm{ex})}{RT} + \nu_{\mathrm{S},j}\ln\left[\frac{\rho_\mathrm{pure}}{\rho_0}\right]\right)\right](V\rho_0)^{\nu_{\mathrm{total,solute},j}} \tag{S7}$$

Using the values of μ_i^0 (computed by quantum chemical calculations[2,4]), μ_i^ex (computed by Monte Carlo simulations[2,4,5]), T, V, and ρ_pure, we can compute the equilibrium constant K'_des for any reaction.

The equilibrium constants can also be defined with the number molecules of each species or the mole fraction of each species:

$$K_j = \prod_{i=1}^{N_\mathrm{species}} X_i^{\nu_{i,j}} \tag{S8}$$

where X_i is the mole fraction of species i. Note that the summation in Eq. (S8) includes the solvent mole fraction and originates from defining the chemical potential of the species using a pure-liquid reference state[6]:

$$\mu_i = \mu_i^* + RT\ln[X_i\gamma_i] \tag{S9}$$

where γ_i is the activity coefficient of species i and μ_i^* is the reference chemical potential of the pure component i in the liquid phase. γ_i is incorporated into K_j in our calculations and assumed constant. To convert K'_j to K_j, we can use:

$$K_j = K'_j \frac{X_\mathrm{s}^{\nu_{\mathrm{s},j}}}{\exp\left[-\nu_{\mathrm{s},j}\left(\frac{1-X_\mathrm{s}}{X_\mathrm{s}}\right)\right]\left(\sum_{i=1}^{N_\mathrm{species}} N_i\right)^{\nu_{\mathrm{total,solute},j}}} \tag{S10}$$

The desired equilibrium constant K_j ($K_{j,\mathrm{des}}$) as a function of μ^0, μ^ex, T, and V can be computed as:

$$K_{j,\mathrm{des}} = \exp\left[-\left(\sum_{i=1}^{N_{\mathrm{species}}} \frac{\nu_{i,j}(\mu_i^0 + \mu_i^{\mathrm{ex}})}{RT} + \nu_{\mathrm{s},j}\ln\left[\frac{\rho_{\mathrm{pure}}}{\rho_0}\right]\right) + \nu_{\mathrm{s},j}\left(\frac{1-X_{\mathrm{s}}}{X_{\mathrm{s}}}\right)\right] \left(\frac{V\rho_0}{\sum_{i=1}^{N_{\mathrm{species}}} N_i}\right)^{\nu_{\mathrm{total,solute},j}} X_{\mathrm{s}}^{\nu_{\mathrm{s},j}} \tag{S11}$$

This means that $K_{j,\mathrm{des}} = K_j$ at equilibrium. $K_{j,\mathrm{des}}$ can be constant or it can be solved iteratively for changing values of X_{s}. In our solver, we solve $K_{j,\mathrm{des}}$ iteratively for changing values of X_{s}. That means we compute new values of X_{s} and $K_{j,\mathrm{des}}$ in every iteration of the solver and this is continued until the difference between the new X_{s} and the old X_{s} no longer changes.

S2 Input File for the Chemical Reaction Equilibrium Solver

In this section, we explain the input file for our chemical reaction equilibrium solver in detail. For this purpose, we used the CO_2/MDEA/water system as a case study. The reactions (Reactions R1–R4 of the main text) and the mass balance equations (Eqs. 11–14 of the main text) involved in this system are shown in the Methods section of the main text. Note that the input file should be in the same directory with **main.py** and **functions.py** for solver to perform properly. An example input file for our solver is:

```
Temperature (K)
313.15
Number of Species
8
C0 (initial guess) / [mol/dm3]
1.0e-10 3.0e-10 1.0e-10 55.0638 2.500E-05 1.0e-10 1.0e-10 2.5
Names of species
HCO3- H3O+  CO3--  H2O  CO2  OH-  MDEAH+  MDEA
```

```
Charges
-1 1 -2 0 0 -1 1 0
Name of the solvent
H2O
Pure Density of the Solvent / [mol/dm3]
55.0638
mu^0 species / [kJ/mol] (only for the calculation of desired equilibrium constants)
0.0 -171.986 0.0 -858.154 0.0 0.0 -6272.15 -6661.2076
mu^ex species / [kJ/mol] (only for the calculation of desired equilibrium constants)
0.0 -779.679 0.0 -26.51 0.0 0.0 -553.792 -31.145
Impose Ptotal and gas composition? (T=True or F=False)
F
Ptotal / [kPa] (only used if Ptotal and gas composition is imposed)
0.0
Gas phase species
CO2
Gas phase composition (only used if Ptotal and gas composition is imposed)
1
mu^ex gases / [kJ/mol]
0.41013
Ctotal,gas / [mol/dm3] (Total concentration of the gases in liquid phase)
2.500E-05 4.059E-05 6.592E-05 1.070E-04 1.738E-04 2.822E-04 4.582E-04 7.441E-04
Number of Reactions
4
Stoichiometry
1 1 0 -2 -1 0 0 0
-1 1 1 -1 0 0 0 0
```

```
0 1 0 -1 0 0 -1 1
0 1 0 -2 0 1 0 0
ln(K) for reactions (if QMMC then computed using K_des expression)
-18.34 -27.55 QMMC -39.21
Number of mass balance equations (excluding charge neutrality)
3
Balances
1 3 5
7 8
2 4 6
```

The lines in the input file represent the following:

- `Temperature`: The absolute temperature.
- `Number of Species`: Number of species in the liquid phase.
- `C0 (initial guess)`: Initial guess of the composition in the liquid phase. This is a list of initial concentrations of species in $\mathrm{mol\,dm^{-3}}$. In our example, we used a lean solvent (only MDEA and water in the solution) as our initial guess. Note that none of the concentrations in the initial guess should be zero (due to the boundaries we use in our solver), instead, one can input a very low concentration. If any concentration in the initial guess is inputted zero or lower than zero (≤ 0), then it is changed by 10^{-10} $\mathrm{mol\,dm^{-3}}$. Although the solver works if the initial guess does not satisfy charge neutrality, we recommend an initial guess that satisfies charge neutrality for quicker results. The solver does not print a warning if charge neutrality is not satisfied by the initial guess and will continue to run.
- `Names of species`: Names of the species in the liquid phase.
- `Charges`: Net charges of the molecules/ions in the liquid phase.

- `Name of the solvent`: The name of the solvent.
- `Pure Density of the Solvent`: Density of the pure solvent in mol dm^{-3}. The name of the solvent and the pure density of the solvent are used to compute the desired equilibrium constants of reactions using Eq. (S11).
- `mu^0 species`: A list of the values of μ_i^0 in kJ mol^{-1} for the species involved in reactions. Only used if the equilibrium constants are computed using Eq. (S11).
- `mu^ex species`: A list of the values of μ_i^{ex} in kJ mol^{-1} for the species involved in reactions. Only used if the equilibrium constants are computed using Eq. (S11).
- `Impose Ptotal and gas composition? (T=True or F=False)`: Are the total gas pressure and the gas composition imposed in the calculation? If True, the solver assumes an infinite gas phase and the speciations are computed for all Ptotal (in kPa) listed in the next line. If the total gas pressure and gas composition are imposed, no mass balance equation is used for the species in the gas phase since there is mass transfer from the infinite gas phase to the liquid phase.
- `Ptotal / [kPa] (only used if Ptotal and gas composition is imposed)`: The list of total gas pressures. Used only if the total gas pressure and gas composition are imposed to compute the partial pressures of the species in the gas phase.
- `Gas phase species`: The names of the species in the gas phase.
- `Gas phase composition (only used if Ptotal and gas composition is imposed)`: The composition of the gas phase. The values in this list are normalized so the values sum up to 1.
- `mu^ex gases / [kJ/mol]`: The values of μ_i^{ex} for the gas phase species in the solvent.
- `Ctotal,gas / [mol/dm3] (Total concentration of the gases in liquid phase)`: A list of concentrations on the gas phase species in the liquid phase in mol dm^{-3}. Only

used if the total gas pressure and gas composition are not imposed.

- `Number of Reactions`: Number of reactions in the liquid phase.
- `Stoichiometry`: The stoichiometric coefficients of all species for each reaction.
- `ln(K) for reactions`: The mole fraction-based equilibrium constants for each reaction in the liquid phase. In case the desired equilibrium constant should be computed using Eq. (S11), the input should be "QMMC".
- `Number of mass balance equations`: Number of mass balance equations.
- `Balances`: A list of species involved in each mass balance equation. For example, the line "1 3 5" shows that the species at the first, third and fifth place in the names line (HCO_3^-, CO_3^{2-}, and CO_2) are included in the first mass balance equation (CO_2 balance).

S3 Computing μ_i^0 and μ_i^{ex} of the Species

S3.1 Computing μ_i^0 using quantum chemistry calculations

Quantum chemistry calculations[2,4] or thermodynamic data sets such as the JANAF tables[7,8] can be used to compute molecular partition functions of isolated molecules. Molecular partition functions can be used to compute heat capacities, internal energies, or chemical potentials of species[2,9]. In this section, we explain how to obtain the standard ideal gas chemical potential of species using the Gaussian09 software[10]. For more detail on molecular partition functions, the reader is referred to Refs.[2,9]. The standard ideal gas chemical potential can be computed using the molecular partition function using[2,9]:

$$\mu_i^0 = -RT \ln\left[\frac{q_{0,i}}{\rho_0 \Lambda_i^3}\right] - D_{0,i} \tag{S12}$$

where μ_i^0 is the standard ideal gas chemical potential of species i, R is the ideal gas constant, T is the absolute temperature, $q_{0,i}$ is the molecular partition function (excluding the translational part) of the molecule with the ground state energy of the molecule is taken as zero[2], ρ_0 is the reference number density of 1 molecule Å^{-3}, Λ_i is the thermal de Broglie wavelength of molecule i, and $D_{0,i}$ is the atomization energy of molecule i, which is the energy required to break all bonds in the molecule[11] ($D_{0,i} > 0$). The atomization energy of molecule i can be computed using[2,10]:

$$D_{0,i} = \sum_{j=1}^{N_{\text{atoms},i}} y_j \varepsilon_{\text{e},j} - \varepsilon_{\text{e},i} - \varepsilon_{\text{ZPE},i} \tag{S13}$$

where $N_{\text{atoms},i}$ is the number of atoms in the molecule i, y_j is the number of atoms of type j in molecule i, $\varepsilon_{\text{e},j}$ is the electronic energy of the atom of type j, $\varepsilon_{\text{e},i}$ is the electronic energy of molecule i, and $\varepsilon_{\text{ZPE},i}$ is the zero point vibrational energy of molecule i (the vibrational energy at the ground state). The definition of μ_i^0 in Eq. (S12) is consistent with the reference state of the chemical potential used in the definitions of Eq. (S2) and Eq. 3 of the main text. As explained in the main text, other definitions of the reference state of the chemical potential are also possible. Eq. (S2) and Eq. (S12) are consistent with Brick-CFCMC[2,5,12].

It should be noted that Gaussian09[10] does not print the electronic energies of individual atoms when the energy of a molecule is computed. Therefore, the electronic energies of individual atoms must be computed separately. It is also important that the zero point energy is included in the electronic energy computed by Gaussian09, so the zero point energy should not be subtracted from the electronic energies of the individual atoms (Eq. (S13)). Also, larger molecules such as MDEA and MEA has many different conformers with different ground state energies. A conformer search must be performed for these type of molecules to obtain the free energies of conformers. Although the conformers with similar free energies can be accounted using a "lumping" procedure[13], we only use the conformer with minimum free energy since the differences between the free energy of the conformer with minimum free energy and the free energies of the other conformers are large ($>> 1\ k_{\text{B}}T$). The standard

state ideal chemical potential of a molecule can be computed using Gaussian09 (with the `Freq` keyword). However, Gaussian09 uses a different reference state ($P_0 = 1\,\text{bar}$) than Brick-CFCMC ($\rho_0 = 1$ molecule Å^{-3}). The standard state ideal gas chemical potential at the Gaussian09 reference state (μ_i^{G}) can be computed using:

$$\mu_i^{\text{G}} = -RT \ln \left[\frac{q_{0,i} k_{\text{B}} T}{P_0 \Lambda_i^3} \right] - D_{0,i} \tag{S14}$$

Also in Eq. (S14), $q_{0,i}$ is used with the ground state energy of the molecule as a reference. The value of the term $\frac{q_{0,i} k_{\text{B}} T}{P_0 \Lambda_i^3}$ and its natural logarithm can be computed by Gaussian09[10] when frequency calculations are enabled (with the `Freq` keyword). The logarithm term in Eq. (S14) is printed by Gaussian09[10] in the thermochemistry section of the output file. The line "`Total V=0`" in the table where "`Q`" is tabulated shows the term $\frac{q_{0,i} k_{\text{B}} T}{P_0 \Lambda_i^3}$ and its natural logarithm. We can use this value tabulated in the thermochemistry section of Gaussian09 output directly in Eq. (S14). To use the values of μ_i^{G} computed by Gaussian09 in CASpy (or Brick-CFCMC[2,5,12]), a conversion is needed to the correct reference state. The conversion from μ_i^{G} (Eq. (S14)) to μ_i^0 (Eq. (S12)) can be performed using:

$$\mu_i^0 = \mu_i^{\text{G}} + RT \ln \left[\frac{k_{\text{B}} T \rho_0}{P_0} \right] \tag{S15}$$

As an example, we will compute the value of μ_i^0 for water at 313.15 K. We optimized and computed free energy of water at 313.15 K using the G4 method. Below the input file to compute the value of μ_i^0 for water using Gaussian09[10] can be found:

```
%Chk=h2o.chk
#p G4 Opt Freq pop=(nbo,esp) Temperature=313.15 Volume

 H2O

0 1
```

```
O          0.00000       -0.11195        0.00000
H         -0.78304        0.49423        0.00000
H          0.78304        0.49423        0.00000
```

We show a small part of the Gaussian09[10] output below.

```
...
-------------------
- Thermochemistry -
-------------------
Temperature   313.150 Kelvin.  Pressure   1.00000 Atm.
Atom     1 has atomic number  8 and mass  15.99491
Atom     2 has atomic number  1 and mass   1.00783
Atom     3 has atomic number  1 and mass   1.00783
Molecular mass:    18.01056 amu.
Principal axes and moments of inertia in atomic units:
                          1         2         3
    Eigenvalues --     2.26052   4.11754   6.37806
          X            0.00000   0.00000   1.00000
          Y            1.00000   0.00000   0.00000
          Z            0.00000   1.00000   0.00000
This molecule is an asymmetric top.
Rotational symmetry number  2.
Rotational temperatures (Kelvin)     38.31594    21.03534    13.57997
Rotational constants (GHZ):         798.37555   438.30579   282.96103
Zero-point vibrational energy      56124.9 (Joules/Mol)
                                  13.41416 (Kcal/Mol)
Vibrational temperatures:   2405.69  5472.96  5621.88
         (Kelvin)
```

```
Zero-point correction=                           0.021377 (Hartree/Particle)
Thermal correction to Energy=                    0.024355
Thermal correction to Enthalpy=                  0.025347
Thermal correction to Gibbs Free Energy=         0.002646
Sum of electronic and zero-point Energies=            -76.404700
Sum of electronic and thermal Energies=               -76.401722
Sum of electronic and thermal Enthalpies=             -76.400730
Sum of electronic and thermal Free Energies=          -76.423431

                    E (Thermal)             CV                S
                     KCal/Mol        Cal/Mol-Kelvin    Cal/Mol-Kelvin
Total                   15.283              6.016             45.490
Electronic               0.000              0.000              0.000
Translational            0.933              2.981             34.852
Rotational               0.933              2.981             10.629
Vibrational             13.416              0.054              0.008
                     Q            Log10(Q)             Ln(Q)
Total Bot       0.693662D-01         -1.158852         -2.668356
Total V=0       0.159514D+09          8.202800         18.887645
Vib (Bot)       0.435059D-09         -9.361452        -21.555540
Vib (V=0)       0.100046D+01          0.000200          0.000461
Electronic      0.100000D+01          0.000000          0.000000
Translational   0.339657D+07          6.531040         15.038276
Rotational      0.469418D+02          1.671560          3.848908

...
```

```
Temperature=              313.150000 Pressure=              1.000000
E(ZPE)=                     0.021065 E(Thermal)=            0.024044
E(CCSD(T))=               -76.207699 E(Empiric)=           -0.027788
DE(Plus)=                  -0.012885 DE(2DF)=              -0.074805
E(Delta-G3XP)=             -0.085401 DE(HF)=               -0.009735
G4(0 K)=                  -76.397249 G4 Energy=           -76.394271
G4 Enthalpy=              -76.393279 G4 Free Energy=      -76.415980
1\1\GINC-C061\Mixed\G4\G4\H2O1\MPOLAT\17-Jun-2022\0\\#p G4 Opt Freq po
p=(nbo,esp) Temperature=313.15 Volume\\H2O\\0,1\O,0,0.,-0.1042712687,0
.\H,0,-0.7563319142,0.4903906343,0.\H,0,0.7563319142,0.4903906343,0.\\
Version=EM64L-G09RevB.01\State=1-A1\MP2/GTBas1=-76.1967582\MP4/GTBas1=
-76.2072004\CCSD(T)/G3Bas1=-76.2076993\MP2/GTBas2=-76.2095143\MP4/GTBa
s2=-76.2200856\MP2/GTBas3=-76.2673009\MP4/GTBas3=-76.2820058\HF/GTLarg
eXP=-76.0573671\MP2/GTLargeXP=-76.3654584\HF/GFHFB1=-76.0648885\HF/GFH
FB2=-76.0666683\G4=-76.3972495\FreqCoord=0.,-0.1970441413,0.,-1.429260
1835,0.926703997,0.,1.4292601835,0.926703997,0.\PG=C02V [C2(O1),SGV(H2
)]\NImag=0\\0.66772935,0.,0.45904202,0.,0.,-0.00003485,-0.33386467,0.1
9890011,0.,0.36695722,0.26249795,-0.22952101,0.,-0.23069903,0.21793357
,0.,0.,0.00001743,0.,0.,-0.00009409,-0.33386467,-0.19890011,0.,-0.0330
9255,-0.03179892,0.,0.36695722,-0.26249795,-0.22952101,0.,0.03179892,0
.01158744,0.,0.23069903,0.21793357,0.,0.,0.00001743,0.,0.,0.00007667,0
.,0.,-0.00009409\\0.,-0.00012296,0.,0.00010199,0.00006148,0.,-0.000101
99,0.00006148,0.\\\@
Job cpu time:  0 days  0 hours  0 minutes 27.4 seconds.
File lengths (MBytes):  RWF=     18 Int=      0 D2E=      0 Chk=      3
Scr=      1
Normal termination of Gaussian 09 at Fri Jun 17 15:41:48 2022.
```

```
78 ---------------------------------------------------------------------
```

The Gaussian09[10] output shows that the natural logarithm of the term $\frac{q_{0,i}(T)k_{\mathrm{B}}T}{P_0\Lambda^3}$ ($P_0 =$ 1 bar) is computed as 18.89 (the line with "`Total V=0`" in thermochemistry section of the output, line 43), the electronic energy (including the zero-point energy) of the molecule is computed as -76.40 Hartree (-2.01×10^5 kJ mol^{-1}) (line 56). From seperate calculations, we computed the electronic energy of a hydrogen atom and an oxygen atom as -0.50 Hartree (-1.31×10^3 kJ mol^{-1}) and -75.05 Hartree (-1.97×10^5 kJ mol^{-1}) at 313.15 K, respectively. Using Eq. (S13), we computed the atomization energy $D_{0,\mathrm{water}}$ as 916.06 kJ mol^{-1}. Using Eq. (S14), and Eq. (S15), we compute the value of μ_i^0 of water as -965.23 kJ mol^{-1} at 313.15 K.

S3.2 Computing μ_i^0 using the JANAF tables

Thermodynamic data sets such as the JANAF tables can also be used to compute μ_i^0 of a molecule. The JANAF tables provide thermodynamic functions and parameters such as the Gibbs free energy, the enthalpy of formation, and heat capacity[7,8] as a function of temperature. As explained in the previous section, we use the ground state energy as the reference state in our calculations, so the Gibbs free energy values should be shifted to the enthalpy at $T = 0$ K. JANAF tables use the same reference state as Gaussian09 ($P_0 = 1$ bar). Therefore, we use the same symbol for the standard state ideal gas chemical potential computed using the JANAF tables as the standard state ideal gas chemical potential computed using Gaussian09 (μ_i^{G}). Note that μ_i^{G} should be converted to μ_i^0 using Eq. (S15) to use the correct reference state in Brick-CFCMC[2,5] or CASpy. The standard ideal gas chemical potential of a molecule can be computed in terms of the entries in JANAF tables using[2]:

$$\mu_i^G = [G_i^0(T) - H_i^0(T_{\mathrm{r}})] - [H_i^0(0\,\mathrm{K}) - H_i^0(T_{\mathrm{r}})] - D_{0,i} \tag{S16}$$

where G_i^0 is the standard ideal gas Gibbs free energy of molecule i, H_i^0 is the standard enthalpy of molecule i, T is the absolute temperature, and T_{r} is the reference temperature

($T_\mathrm{r} = 298.15\,\mathrm{K}$ for the JANAF tables). The terms $-[G_i^0(T) - H_i^0(T_\mathrm{r})]/T$ and $[H_i^0(T) - H_i^0(T_\mathrm{r})]$ reported in the JANAF tables can be used to compute the terms $[G_i^0(T) - H_i^0(T_\mathrm{r})]$ and $[H_i^0(0\,\mathrm{K}) - H_i^0(T_\mathrm{r})]$ in Eq. (S16). The atomization energy $D_{0,i}$ can also be computed using the JANAF tables using the difference between the enthalpy of formation of the molecule and the enthalpy of formation of the individual atoms[2]:

$$D_{0,i} = \sum_{j=1}^{N_{\mathrm{atoms},i}} y_j \Delta_f H_j^0(0\,\mathrm{K}) - \Delta_f H_i^0(0\,\mathrm{K}) \tag{S17}$$

where $N_{\mathrm{atoms},i}$ is the number of atoms in molecule i, y_j denotes the number of atoms of type j in molecule i, and $\Delta_f H^0$ is the enthalpy of formation as tabulated in the JANAF tables. As an example, we compute μ_i^0 of water at 313.15 K. The terms $-[G_i^0(T) - H_i^0(T_\mathrm{r})]/T$ and $[H_i^0(0\,\mathrm{K}) - H_i^0(T_\mathrm{r})]$ are reported as $188.87\,\mathrm{J\,K^{-1}\,mol^{-1}}$ and $-9.904\,\mathrm{kJ\,mol^{-1}}$ for water, respectively. Also, $\Delta_f H^0_{\mathrm{H_2O}}(0\,\mathrm{K})$, $\Delta_f H^0_{\mathrm{H}}(0\,\mathrm{K})$, and $\Delta_f H^0_{\mathrm{O}}(0\,\mathrm{K})$ are listed in the JANAF tables as $-238.921\,\mathrm{kJ\,mol^{-1}}$, $216.035\,\mathrm{kJ\,mol^{-1}}$, and $246.79\,\mathrm{kJ\,mol^{-1}}$, respectively. Using the values of $\Delta_f H_i^0(0\,\mathrm{K})$ and Eq. (S17), we computed the atomization energy $D_{0,\mathrm{water}}$ as $917.84\,\mathrm{kJ\,mol^{-1}}$. The $D_{0,\mathrm{water}}$ computed using quantum chemical calculations ($916.06\ \mathrm{kJ\,mol^{-1}}$) is in excellent agreement with the $D_{0,\mathrm{water}}$ computed using the JANAF tables. More accurate values of $D_{0,i}$ with more accurate quantum chemistry composite methods can be obtained, as the value of μ_i^0 is sensitive to the value of $D_{0,i}$ (Eq. (S12)). Using Eq. (S16) and Eq. (S15), we computed μ_i^0 of water as -986.83 $\mathrm{kJ\,mol^{-1}}$. The difference between the value of μ_i^0 computed using quantum chemistry (-965.23 $\mathrm{kJ\,mol^{-1}}$, see the previous section) and the value of μ_i^0 computed using JANAF tables is ca. 21 $\mathrm{kJ\,mol^{-1}}$ which is well beyond the chemical accuracy of $4.18\,\mathrm{kJ\,mol^{-1}}$ ($1\,\mathrm{kcal\,mol^{-1}}$). More accurate values of μ_i^0 can be obtained using other quantum chemistry composite methods which is beyond the scope of this manuscript[14].

S3.3 Computing μ_i^{ex} using Brick-CFCMC

Brick-CFCMC is an open source Monte Carlo simulation software to compute the phase and reaction equilibria[2,5]. Brick-CFCMC uses the efficient Continuous Fractional Component Monte Carlo (CFCMC) method[2,5,12,15–17] for molecule insertions and deletions which allows us to compute partial molar properties and μ_i^{ex}. The CFCMC method uses a so-called "fractional" molecule group to compute partial molar properties and the values of μ_i^{ex}. An interaction scaling factor λ is used to scale the interactions of the fractional molecule group with the surrounding molecules. At $\lambda = 1$, the fractional molecule group has full interactions with the surrounding molecules while at $\lambda = 0$, the fractional molecule group has no interactions with the surrounding molecules. To compute μ_i^{ex}, we can use two different methods implemented in Brick-CFCMC. The first method is the "probability" route and it uses the probability distribution of the interaction scaling factor λ at $\lambda = 1$ and $\lambda = 0$ to compute μ_i^{ex}:[2,5]

$$\mu_i^{\text{ex}} = -RT\ln\left[\frac{p(\lambda_i = 1)}{p(\lambda_i = 0)}\right] \tag{S18}$$

where $p(\lambda_i = 1)$ and $p(\lambda_i = 0)$ are the probabilities of $\lambda = 1$ and $\lambda = 0$, respectively. This method requires a flat distribution of (observed) λ and this is obtained using a biasing function[2,5,12,15–17]. A rule of thumb for the flat distribution of λ is that the difference between the maximum and minimum probabilities should be lower than 20%. The second method is thermodynamic integration[5]. In thermodynamic integration, we use the average value of the derivative of the potential energy with respect to λ, $\left\langle \frac{\partial U}{\partial \lambda} \right\rangle$, and compute μ_i^{ex} using[4,5]:

$$\mu_i^{\text{ex}} = \int_0^1 \mathrm{d}\lambda \left\langle \frac{\partial U}{\partial \lambda} \right\rangle_{NPT} \tag{S19}$$

It is very challenging to obtain a flat probability distribution of λ in a single simulation for large and/or polar molecules[5]. As mentioned in the methodology section of the main text, we need to compute μ_i^{ex} of ionic and/or large molecules such as the hydronium ion (H_3O^+) and the protonated MDEA ion (MDEAH^+). Using thermodynamic integration, a

flat probability distribution of λ is not required since $\left\langle \frac{\partial U}{\partial \lambda} \right\rangle$ term can be computed from independent MC simulations at different and fixed λ values. Therefore, the thermodynamic integration as implemented in Brick-CFCMC is used to compute μ_i^{ex} in this study. The term $\left\langle \frac{\partial U}{\partial \lambda} \right\rangle$ can only be computed for one charge-neutral fractional group[2,5]. The fractional group can consist of multiple molecules or ions. In this study, we computed the μ_i^{ex} of the reactants and reaction products of $MDEAH^+$ dissociation reaction. As the fractional group should be charge-neutral, an HCO_3^- ion is added to the fractional groups. For example, μ_i^{ex} of the reactants of the $MDEAH^+$ dissociation reaction (reaction R3 of the main text) can be computed using a fractional group consisting of one $MDEAH^+$ ion, one H_2O molecule, and one HCO_3^- ion. μ_i^{ex} of reaction products of $MDEAH^+$ dissociation reaction can be computed using a fractional group consisting of one MDEA molecule, one H_3O^+ ion, and one HCO_3^- ion. In this way, we can compute the difference between μ_i^{ex} of $MDEAH^+ + H_2O$ and μ_i^{ex} of $MDEA + H_3O^+$ (because the excess chemical potential of HCO_3^- cancels out).

Table S1: The list of fractional groups for which we computed μ_i^{ex} and the molecules included in the fractional groups.

Fractional group name	Molecules or ions included in fractional group
MDEA	MDEA
H_2O	H_2O
CO_2	CO_2
H_2S	H_2S
$MDEAH^+ + HCO_3^-$	$MDEAH^+$ and HCO_3^-
$H_3O^+ + HCO_3^-$	H_3O^+ and HCO_3^-

S4 Simulation Details

For thermodynamic integration, we used 50 equidistant and fixed values of λ with two additional simulations at $\lambda = 10^{-6}$ and $\lambda = 1 - 10^{-6}$ to increase the accuracy of thermodynamic integration. In these simulations, a simulation box of 300 water molecules and a single fractional group (see Table S1) was used. Initial configurations for these simulations were created

by randomly inserting molecules to a simulation box with a length of 20.8 Å. The random insertion of molecules into the simulation box causes atomic overlaps and these overlaps were eliminated by 10^3 initialization cycles in which only single molecule translation and single molecule rotation trial moves were performed. The initialization cycles were followed by 10^6 equilibration cycles and 10^6 production cycles. For every MC cycle, N MC trial moves were performed where N is the number of molecules in the simulation box. In the equilibration and production cycles, single molecule translations (48.49%), single molecule rotations (48.49%), hybrid MD/MC translations (0.01%), hybrid MD/MC rotations (0.01%)[5], volume changes (1%), bond bending (1%), and torsion (1%) trial moves were performed with fixed probabilities. The time step and the number of time steps for hybrid MD/MC trial moves were set as 5 fs and 2 MD cycles, respectively. In the MC simulations, a 10 Å distance was used as the LJ cutoff distance and analytic tail corrections[18] were applied. The electrostatic potential was computed using the Ewald summation[19].We set the Ewald summation parameters to 10 Å and $0.32\,\text{Å}^{-1}$ for the cutoff radius and the damping parameter, respectively. After the MC simulations, a spline was fitted to the $\left\langle \frac{\partial U}{\partial \lambda} \right\rangle$ values as a function of λ and the spline was integrated from $\lambda = 0$ to $\lambda = 1$ to compute μ_i^{ex} (Eq. (S19)). The computed values of μ_i^{ex} were not corrected for the finite-size effects[20,21] since the corrections were found insignificant.

For the computation of μ_i^{ex} of all species, the TIP3P[22] force field was used for (rigid) water molecules. We used the TIP3P[22] force field for water because μ_i^{ex} of water computed using TIP3P force field agrees much better with μ_i^{ex} measured using empirical data than the TIP4P or TIP5P force fields[23]. For MDEA, $MDEAH^+$, and (rigid) HCO_3^-, we used either the General Amber Force Field (GAFF)[24] or the OPLS-AA force field[25,26]. The electrostatic potential energy grid of the molecules were computed using the Merz-Kollman scheme[27] in Gaussian09[10] at HF/6-31G* level of theory. The electrostatic potentials computed by Gaussian09 were then fitted with Antechamber package[28] using a two-step Restrained Electrostatic Potential Surface (RESP) fitting method. The RESP fitted point charges were used

as the point charges of GAFF. The parameters for the OPLS-AA force field with 1.14*CM1A point charges were generated using the LibParGen web server[29]. We used the TraPPE[30] and the force field from Kristóf and Lizsi[31] for CO_2 and H_2S molecules, respectively. All force field parameters used in this study are listed in tables S1–S17. The force field parameters for unlike LJ interactions were computed using Lorentz-Berthelot mixing rules[18] except for the TraPPE CO_2 and TIP3P water molecules. The LJ interactions between TraPPE CO_2 and TIP3P water molecules were computed using the optimized potential for CO_2/H_2O mixtures developed by Orozco et al.[32] (Table S6).

For flexible molecules (MDEA and $MDEAH^+$), the bond lengths are fixed. For flexible molecules, the bending potential was computed using[2]:

$$U_{\text{Bending}} = \frac{K}{2}(\theta - \theta_0)^2 \tag{S20}$$

where K is the bending constant, θ is the bending angle, θ_0 is the bending angle at equilibrium. For the GAFF[24], the torsion potential was computed using[2,33]:

$$U_{\text{Torsion}} = \sum_{i=0}^{5} p_i \cos^i(\phi) \tag{S21}$$

where $p_0..p_5$ are the torsion constants and ϕ is the torsion angle. For the OPLS-AA force field, the torsion potential was computed using[2,25,26]:

$$U_{\text{Torsion}} = \frac{K_1}{2}(1+\cos\phi) + \frac{K_2}{2}(1-\cos2\phi) + \frac{K_3}{2}(1+\cos3\phi) + \frac{K_4}{2}(1-\cos4\phi) \tag{S22}$$

where $K_1..K_4$ are the torsion constants and ϕ is the torsion angle.

Table S2: The atom types and coordinates of the TIP3P[22] water molecule.

Atom type	x / [Å]	y / [Å]	z / [Å]
O	0.000	0.000	0.000
H	-0.757	0.586	0.000
H	0.757	0.586	0.000

Table S3: Non-bonded interaction parameters for water. The TIP3P force field[22] was used.

Atom	ϵ/k_B / [K]	σ / [Å]	q / [e^-]
O	76.5414	3.15061	-0.834
H	0.00000	0.00000	0.417

Table S4: The atom types and coordinates of the TraPPE[30] CO_2 molecule.

Atom type	x / [Å]	y / [Å]	z / [Å]
C	1.16	0.00	0.00
O	0.00	0.00	0.00
O	2.32	0.00	0.00

Table S5: Non-bonded interaction parameters for carbon dioxide. The TraPPE force field[30] was used.

Atom	ϵ/k_B / [K]	σ / [Å]	q / [e^-]
O	79.0	3.05	-0.35
C	27.0	2.80	0.70

Table S6: Non-bonded interaction parameters between carbon dioxide and water. The optimized potential developed by Orozco et al.[32] was used.

Atoms	ϵ/k_B / [K]	σ / [Å]
$O_{CO_2} - O_{H_2O}$	79.14	3.058
$C_{CO_2} - O_{H_2O}$	53.04	3.052

Table S7: The atom types and coordinates of the H_2S molecule. The force field from Kristóf and Lizsi[31] was used.

Atom type	x / [Å]	y / [Å]	z / [Å]
S	0.000	0.000	0.000
H	0.964	-0.931	0.000
H	-0.964	-0.931	0.000
X	0.000	-0.186	0.000

Table S8: Non-bonded interaction parameters for hydrogen sulfide. The force field from Kristóf and Lizsi[31] was used. "X" designates the dummy charge in the force field.

Atom	ϵ/k_B / [K]	σ / [Å]	q / [e^-]
S	250.0	3.73	0.40
H	0.000	0.00	0.25
X	0.000	0.00	-0.90

Table S9: The atom types and coordinates of the rigid HCO_3^- ion.

Atom type	x / [Å]	y / [Å]	z / [Å]
OH	-0.724	-0.764	0.0
O	1.521	-0.541	0.0
O	0.182	1.301	0.0
C	0.446	0.096	0.0
HO	-0.306	-1.632	0.0

Table S10: Non-bonded interaction parameters for HCO_3^-. For Lennard-Jones (LJ) interactions, the General Amber Force Field (GAFF)[24] was used. For electrostatic interactions, two-step RESP fitted point charges[28] were used. The point charges listed in this table sum up to -1.

Atom	ϵ/k_B / [K]	σ / [Å]	q / [e^-]
OH	105.8792	3.06469	-0.728557
O	105.6775	2.95992	-0.826268
C	43.27747	3.39967	1.057315
HO	0.000000	0.00000	0.323778

Table S11: The atom types and coordinates of the rigid H_3O^+ ion[34].

Atom type	x / [Å]	y / [Å]	z / [Å]
OH	-0.00044	-0.00003	0.04871
HO	-0.01990	-0.94884	-0.19579
HO	0.82552	0.44398	-0.23683
HO	-0.80407	0.47899	-0.24304

Table S12: Non-bonded interaction parameters for H_3O^+. For H_3O^+ ions, the force field developed by Noroozi and Smith[34] was used. The point charges listed in this table sum up to 1.

Atom	ϵ/k_B / [K]	σ / [Å]	q / [e^-]
OH	76.54135	3.15061	-1.2797
HO	1.000000	1.00000	0.7599

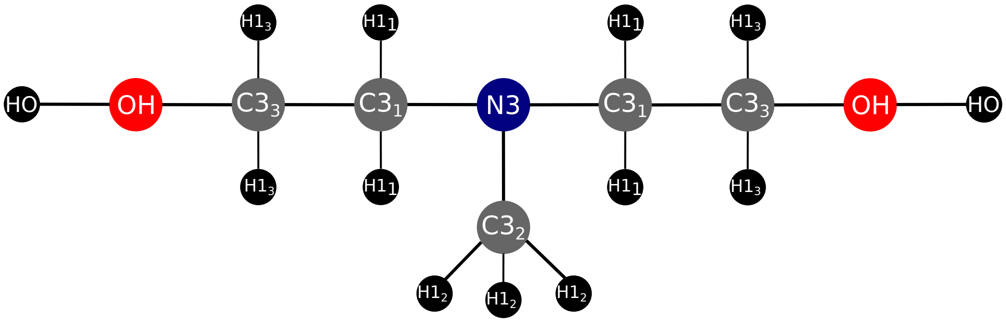

Figure S1: Schematic representation showing atom type designations of the MDEA molecule for the GAFF[24].

Table S13: Non-bonded interaction parameters for MDEA. For Lennard-Jones (LJ) interactions, the General Amber Force Field (GAFF)[24] was used. For electrostatic interactions, two-step RESP fitted point charges[28] were used. The point charges of the MDEA molecule sum up to zero. Fig. S1 shows the atom types designation of MDEA for the GAFF[24].

Atom	ϵ/k_B / [K]	σ / [Å]	q / [e^-]
OH	105.8789	3.06647	-0.715283
N3	85.54849	3.25000	-0.495858
$C3_1$	55.05302	3.39967	0.024816
$C3_2$	55.05302	3.39967	-0.041777
$C3_3$	55.05302	3.39967	0.290555
$H1_1$	7.900655	2.47135	0.060612
$H1_2$	7.900655	2.47135	0.059755
$H1_3$	7.900655	2.47135	0.015339
HO	0.000000	0.00000	0.427195

Table S14: Bond lengths of the MDEA molecule with the General Amber Force field (GAFF)[24]. The bond lengths are fixed in MC simulations.

Bond	Bond length / [Å]
OH_HO	0.96
OH_$C3_3$	1.42
N3_$C3_1$	1.46
N3_$C3_2$	1.46
$C3_1$_$H1_1$	1.09
$C3_1$_$C3_3$	1.53
$C3_2$_$H1_2$	1.09
$C3_3$_$H1_3$	1.09

Table S15: Bending potential parameters, the equilibrium angle (θ_0) and the bending constant (K) of the MDEA molecule for the GAFF[24]. The bending potential was computed using Eq. (S20). Fig. S1 shows the atom types designation of MDEA for the GAFF[24].

Bending	θ_0 / [°]	K/k_B / [K rad^{-2}]
$OH\_C3_3\_C3_1$	110.19	67935.6
$OH\_C3_3\_H1_3$	110.26	51228.3
$N3\_C3_1\_C3_3$	111.04	66426.1
$N3\_C3_1\_H1_1$	109.88	49819.9
$N3\_C3_2\_H1_2$	109.88	49819.9
$C3_1\_N3\_C3_2$	112.35	64211.9
$C3_1\_C3_3\_H1_3$	109.56	46700.0
$C3_3\_OH\_HO$	107.26	47705.5
$C3_3\_C3_1\_H1_1$	109.56	46700.0
$H1_1\_C3_1\_H1_1$	108.46	39453.5
$H1_2\_C3_2\_H1_2$	108.46	39453.5
$H1_3\_C3_3\_H1_3$	108.46	39453.5

Table S16: Torsion potential parameters of the MDEA molecule for the GAFF[24]. The torsion potential was computed using Eq. (S21). Fig. S1 shows the atom types designation of MDEA for the GAFF[24]. The values of p_4 and p_5 are zero for all torsions in the MDEA molecule.

Torsion	p_0/k_B / [K]	p_1/k_B / [K]	p_2/k_B / [K]	p_3/k_B / [K]
$N3\_C3_1\_C3_3\_OH$	78.28	234.84	0.0	-313.12
$OH\_C3_3\_C3_1\_H1_1$	125.81	-125.81	0.0	0.0
$N3\_C3_1\_C3_3\_H1_3$	78.28	234.84	0.0	-313.12
$C3_1\_N3\_C3_1\_C3_3$	634.07	452.9	-483.10	-603.87
$C3_1\_N3\_C3_1\_H1_1$	150.97	452.9	0.0	-603.87
$C3_1\_N3\_C3_2\_H1_2$	150.97	452.9	0.0	-603.87
$C3_1\_C3_3\_OH\_HO$	206.32	115.74	0.0	-322.06
$C3_2\_N3\_C3_1\_C3_3$	634.07	452.9	-483.10	-603.87
$C3_2\_N3\_C3_1\_H1_1$	150.97	452.9	0.0	-603.87
$H1_1\_C3_1\_C3_3\_H1_3$	78.28	234.84	0.0	-313.12
$H1_3\_C3_3\_OH\_HO$	83.87	251.61	0.0	-335.48
$HO\_OH\_C3_3\_H1_3$	83.87	251.61	0.0	-335.48

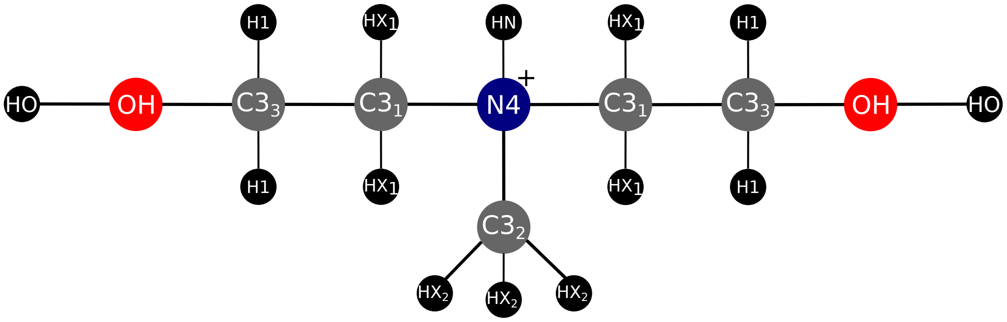

Figure S2: Schematic representation showing atom type designations of the MDEAH$^+$ ion for the GAFF[24].

Table S17: Non-bonded interaction parameters for MDEAH$^+$. For Lennard-Jones (LJ) interactions, the General Amber Force Field (GAFF)[24] was used. For electrostatic interactions, two-step RESP fitted point charges[28] were used. The point charges of the MDEAH$^+$ ion sum up to -1. Fig. S2 shows the atom types designation of MDEAH$^+$ for the GAFF[24].

Atom	ϵ/k_B / [K]	σ / [Å]	q / [e^-]
OH	105.8789	3.06647	-0.700894
N4	85.54849	3.25000	0.168263
$C3_1$	55.05302	3.39967	-0.144533
$C3_2$	55.05302	3.39967	-0.606302
$C3_3$	55.05302	3.39967	0.274300
HX_1	7.900655	2.47135	0.124590
HX_2	7.900655	2.47135	0.228587
H1	7.900655	2.47135	0.033983
HN	7.900655	2.47135	0.313387
HO	0.000000	0.00000	0.473426

Table S18: Bond lengths of the MDEAH$^+$ ion with the General Amber Force field (GAFF)[24]. The bond lengths are fixed in MC simulations.

Bond	Bond length / [Å]
OH_HO	0.96
OH_$C3_3$	1.42
N4_$C3_1$	1.51
N4_$C3_2$	1.51
$C3_1$_HX_1	1.09
$C3_1$_$C3_3$	1.53
$C3_1$_HX_1	1.09
$C3_2$_HX_2	1.09
$C3_3$_H1	1.10
N4_HN	1.03

Table S19: Bending potential parameters, the equilibrium angle (θ_0) and the bending constant (K) of the $MDEAH^+$ ion for the GAFF[24]. The bending potential was computed using Eq. (S20). Fig. S2 shows the atom types designation of $MDEAH^+$ for the GAFF[24].

Bending	θ_0 / [°]	K/k_B / [K rad^{-2}]
OH_$C3_3$_$C3_1$	110.19	67935.6
OH_$C3_3$_H1	110.26	51228.3
N4_$C3_1$_$C3_3$	114.21	64618.8
N4_$C3_1$_HX_1	108.01	48913.0
N4_$C3_2$_HX_2	108.01	48913.0
$C3_1$_N4_$C3_1$	109.66	63004.3
$C3_1$_N4_$C3_2$	109.66	63004.3
$C3_1$_N4_HN	110.11	46196.0
$C3_1$_$C3_3$_H1	109.56	46700.0
$C3_2$_N4_HN	110.11	46196.0
$C3_3$_OH_HO	107.26	47705.5
$C3_3$_$C3_1$_HX_1	110.56	46497.9
HX_1_$C3_1$_HX_1	109.75	39453.5
HX_2_$C3_2$_HX_2	109.75	39453.5
H1_$C3_3$_H1	108.46	39453.5

Table S20: Torsion potential parameters of the $MDEAH^+$ ion for the GAFF[24]. The torsion potential was computed using Eq. (S21). Fig. S2 shows the atom types designation of $MDEAH^+$ for the GAFF[24]. The values of p_4 and p_5 are zero for all torsions in the $MDEAH^+$ ion.

Torsion	p_0/k_B / [K]	p_1/k_B / [K]	p_2/k_B / [K]	p_3/k_B / [K]
N4_$C3_1$_$C3_3$_OH	72.47	217.39	1308.39	-289.86
OH_$C3_3$_$C3_1$_HX_1	78.28	234.84	0.0	-313.12
N4_$C3_1$_$C3_3$_H1	78.28	234.84	0.0	-313.12
$C3_1$_N4_$C3_1$_$C3_3$	78.28	234.84	0.0	-313.12
$C3_1$_N4_$C3_1$_HX_1	78.28	234.84	0.0	-313.12
$C3_1$_N4_$C3_1$_HX_2	78.28	234.84	0.0	-313.12
$C3_1$_$C3_3$_OH_HO	206.32	115.74	0.0	-322.06
$C3_2$_N4_$C3_1$_$C3_3$	78.28	234.84	0.0	-313.12
$C3_2$_N4_$C3_1$_HX_1	78.28	234.84	0.0	-313.12
HN_N4_$C3_1$_$C3_3$	78.28	234.84	0.0	-313.12
H1_$C3_3$_$C3_1$_HX_1	78.28	234.84	0.0	-313.12
HX_1_$C3_1$_N4_HN	78.28	234.84	0.0	-313.12
HX_1_$C3_2$_N4_HN	78.28	234.84	0.0	-313.12
H1_$C3_3$_OH_HO	83.87	251.61	0.0	-335.48
$C3_1$_$C3_3$_OH_HO	206.32	115.74	0.0	-322.06

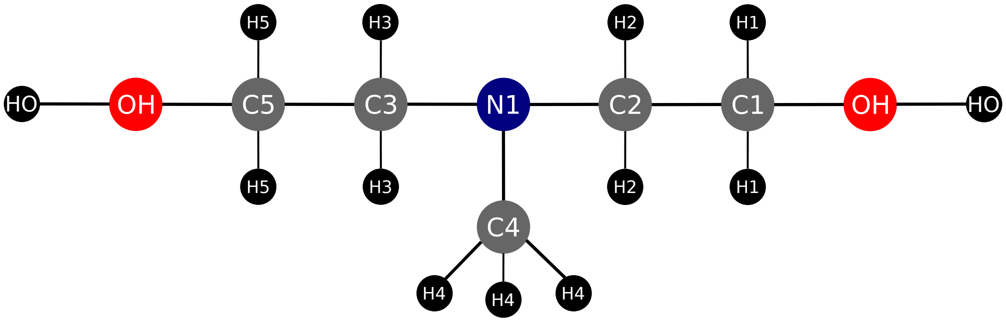

Figure S3: Schematic representation showing atom type designations of the MDEA molecule for the OPLS-AA force field[25,26].

Table S21: Non-bonded interaction parameters for MDEA. For Lennard-Jones (LJ) interactions, the OPLS-AA force field[25,26] was used. For electrostatic interactions, 1.14*CM1A point charges[35] were used. The point charges of the MDEA molecule sum up to zero. Fig. S3 shows the atom types designation of MDEA for the OPLS-AA force field[25,26].

Atom	ϵ/k_B / [K]	σ / [Å]	q / [e^-]
HO	0.000000	0.00	0.40770
OH	85.548491	3.12	-0.58335
C1	33.212943	3.50	0.00440
C2	33.212943	3.50	-0.01000
C3	33.212943	3.50	0.02920
C4	33.212943	3.50	-0.02180
C5	33.212943	3.50	0.00700
H1	15.096792	2.50	0.10210
H2	15.096792	2.50	0.08810
H3	15.096792	2.50	0.09900
H4	15.096792	2.50	0.08300
H5	15.096792	2.50	0.08090
N1	85.548491	3.30	-0.64670

Table S22: Bond lengths of the MDEA molecule with the OPLS-AA force field[25,26]. The bond lengths are fixed in MC simulations.

Bond	Bond length / [Å]
HO_OH	0.96
OH_C1	1.42
C1_C2	1.53
C1_H1	1.10
C2_N1	1.46
C2_H2	1.11
N1_C3	1.46
N1_C4	1.46
C3_C5	1.53
C3_H3	1.11
C4_H4	1.11
C5_H5	1.10
C5_OH	1.42
OH_HO	0.96

Table S23: Bending potential parameters, the equilibrium angle (θ_0) and the bending constant (K) of the MDEA molecule for the OPLS-AA force field[25,26]. The bending potential was computed using Eq. (S20). Fig. S3 shows the atom types designation of MDEA for the OPLS-AA force field[25,26].

Bending	θ_0 / [°]	K/k_B / [K rad^{-2}]
HO_OH_C1	108.50	55354.9
OH_C1_C2	109.50	50322.6
OH_C1_H1	109.50	35225.8
C1_C2_N1	109.47	56562.7
C1_C2_H2	110.70	37742.0
C2_N1_C3	107.20	52134.2
C2_N1_C4	107.20	52134.2
N1_C3_C5	109.47	56562.7
N1_C3_H3	109.50	35225.8
N1_C4_H4	109.50	35225.8
C3_C5_OH	109.50	50322.6
C3_C5_H5	110.70	37742.0
C5_OH_HO	108.50	55354.9
H4_C4_H4	107.80	33212.9
H3_C3_H3	107.80	33212.9
C5_C3_H3	110.70	37742.0
N1_C2_H2	109.50	35225.8
H4_C4_H4	107.80	33212.9
C5_C3_H3	110.70	37742.0
N1_C2_H2	109.50	35225.8
H5_C5_H5	107.80	33212.9
OH_C5_H5	109.50	35225.8
C2_C1_H1	110.70	37742.0
OH_C5_H5	109.50	35225.8
C2_C1_H1	110.70	37742.0
H2_C2_H2	107.80	33212.9
C3_N1_C4	107.20	52134.2
H1_C1_H1	107.80	33212.9
H4_C4_H4	107.80	33212.9

Table S24: Torsion potential parameters of the MDEA molecule for the OPLS-AA force field[25,26]. The torsion potential was computed using Eq. (S22). Fig. S3 shows the atom types designation of MDEA for the OPLS-AA force field[25,26].

Torsion	K_1/k_B / [K]	K_2/k_B / [K]	K_3/k_B / [K]	K_4/k_B / [K]
C5_C3_N1_C2	215.17	420.00	64.47	-699.51
C5_C3_N1_C4	215.17	420.00	64.47	-699.51
C2_C1_OH_HO	-53.40	461.01	87.56	-495.17
C3_N1_C2_C1	215.17	420.00	64.47	-699.51
C4_N1_C2_H2	140.96	422.76	0.00	-563.60
C3_N1_C2_H2	140.96	422.76	0.00	-563.60
H5_C5_C3_H3	75.53	226.48	0.00	-301.89
H2_C2_C1_H1	75.53	226.48	0.00	-301.89
H5_C5_C3_H3	75.53	226.48	0.00	-301.89
H5_C5_C3_N1	-492.64	611.95	356.73	-476.04
H2_C2_C1_OH	117.75	353.24	0.00	-470.99
H4_C4_N1_C2	140.96	422.76	0.00	-563.60
H3_C3_N1_C4	140.96	422.76	0.00	-563.60
H4_C4_N1_C3	140.96	422.76	0.00	-563.60
H3_C3_N1_C2	140.96	422.76	0.00	-563.60
H4_C4_N1_C2	140.96	422.76	0.00	-563.60
H1_C1_OH_HO	88.52	265.69	0.00	-354.33
HO_OH_C5_C3	-53.40	461.01	87.56	-495.17
HO_OH_C5_H5	88.52	265.69	0.00	-354.33
N1_C2_C1_H1	-492.64	611.95	356.73	-476.04
N1_C2_C1_OH	2012.91	-2012.91	0.00	0.00
OH_C5_C3_H3	117.75	353.24	0.00	-470.99
OH_C5_C3_N1	2012.91	-2012.91	0.00	0.00

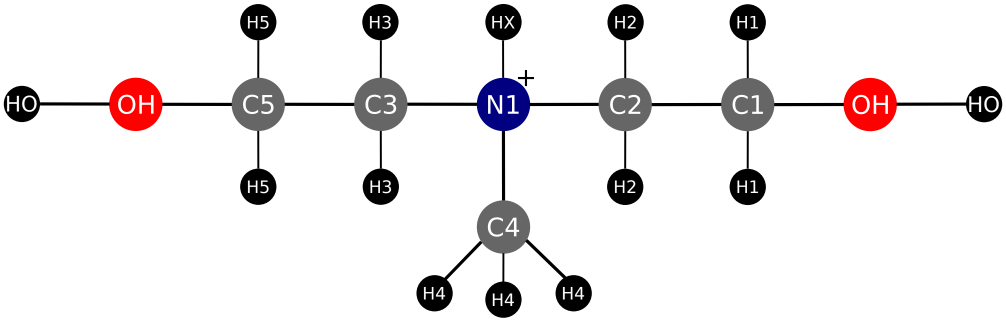

Figure S4: Schematic representation showing atom type designations of the $MDEAH^+$ ion for the OPLS-AA force field[25,26].

Table S25: Non-bonded interaction parameters for $MDEAH^+$. For Lennard-Jones (LJ) interactions, the OPLS-AA force field[25,26] was used. For electrostatic interactions, 1.14*CM1A point charges[35] were used. The point charges of the $MDEAH^+$ ion sum up to -1. Fig. S4 shows the atom types designation of $MDEAH^+$ for the OPLS-AA force field[25,26].

Atom	ϵ/k_{B} / [K]	σ / [Å]	q / [e^-]
HO	0.000000	0.00	0.40485
OH	85.548491	3.12	-0.54030
C1	33.212943	3.50	-0.00870
C2	33.212943	3.50	-0.10110
C3	33.212943	3.50	-0.10380
C4	33.212943	3.50	-0.15560
C5	33.212943	3.50	-0.00980
H1	15.096792	2.50	0.11010
H2	15.096792	2.50	0.14220
H3	15.096792	2.50	0.14330
H4	15.096792	2.50	0.13400
H5	15.096792	2.50	0.10990
HX	0.000000	0.00	0.36670
N1	85.548491	3.25	-0.12980

Table S26: Bond lengths of the $MDEAH^+$ ion with the OPLS-AA force field[25,26]. The bond lengths are fixed in MC simulations.

Bond	Bond length / [Å]
HO_OH	0.96
OH_C1	1.42
C1_C2	1.53
C1_H1	1.10
C2_N1	1.51
C2_H2	1.09
N1_C3	1.51
N1_C4	1.50
N1_HX	1.03
C3_C5	1.53
C3_H3	1.09
C4_H4	1.09
C5_OH	1.41
C5_H5	1.10
OH_HO	0.96

Table S27: Bending potential parameters, the equilibrium angle (θ_0) and the bending constant (K) of the $MDEAH^+$ ion for the OPLS-AA force field[25,26]. The bending potential was computed using Eq. (S20). Fig. S4 shows the atom types designation of $MDEAH^+$ for the OPLS-AA force field[25,26].

Bending	θ_0 / [°]	K/k_B / [K rad^{-2}]
HO_OH_C1	108.50	55354.9
OH_C1_C2	109.50	50322.6
OH_C1_H1	109.50	35225.8
C1_C2_N1	111.20	80516.2
C1_C2_H2	110.70	37742.0
C2_N1_C3	113.00	50322.6
C2_N1_C4	113.00	50322.6
C2_N1_HX	107.64	32357.4
N1_C3_C5	111.20	80516.2
N1_C3_H3	109.50	35225.8
N1_C4_H4	109.50	35225.8
C3_C5_OH	109.50	50322.6
C3_C5_H5	110.70	37742.0
C5_OH_HO	108.50	55354.9
C4_N1_HX	107.64	32357.4
C5_C3_H3	110.70	37742.0
OH_C5_H5	109.50	35225.8
H4_C4_H4	107.80	33212.9
N1_C2_H2	109.50	35225.8
H5_C5_H5	107.80	33212.9
C3_N1_HX	107.64	32357.4
C2_C1_H1	110.70	37742.0
C5_C3_H3	110.70	37742.0
H4_C4_H4	107.80	33212.9
H2_C2_H2	107.80	33212.9
C3_N1_C4	113.00	50322.6
H1_C1_H1	107.80	33212.9
OH_C5_H5	109.50	35225.8
H4_C4_H4	107.80	33212.9
H3_C3_H3	107.80	33212.9

Table S28: Torsion potential parameters of the $MDEAH^+$ ion for the OPLS-AA force field[25,26]. The torsion potential was computed using Eq. (S22). Fig. S4 shows the atom types designation of $MDEAH^+$ for the OPLS-AA force field[25,26].

Torsion	K_1/k_B / [K]	K_2/k_B / [K]	K_3/k_B / [K]	K_4/k_B / [K]
C5_C3_N1_C4	365.87	-162.49	62.42	-265.69
C5_C3_N1_C2	365.87	-162.49	62.42	-265.69
C5_C3_N1_HX	87.32	261.96	0.00	-349.28
C2_C1_OH_HO	-53.40	461.01	87.56	-495.17
C4_N1_C2_C1	365.87	-162.49	62.42	-265.69
C3_N1_C2_C1	365.87	-162.49	62.42	-265.69
C4_N1_C2_H2	76.01	227.92	0.00	-303.93
C3_N1_C2_H2	76.01	227.92	0.00	-303.93
HX_N1_C2_C1	87.32	261.96	0.00	-349.28
HX_N1_C2_H2	65.67	197.01	0.00	-262.68
H5_C5_C3_H3	75.53	226.48	0.00	-301.89
H2_C2_C1_H1	75.53	226.48	0.00	-301.89
H5_C5_C3_H3	75.53	226.48	0.00	-301.89
H5_C5_C3_N1	96.58	289.86	0.00	-386.44
H2_C2_C1_OH	117.75	353.24	0.00	-470.99
H4_C4_N1_C2	76.01	227.92	0.00	-303.93
H4_C4_N1_C3	76.01	227.92	0.00	-303.93
H3_C3_N1_C4	76.01	227.92	0.00	-303.93
H3_C3_N1_C2	76.01	227.92	0.00	-303.93
H4_C4_N1_C2	76.01	227.92	0.00	-303.93
H3_C3_N1_C4	76.01	227.92	0.00	-303.93
H4_C4_N1_HX	65.67	197.01	0.00	-262.68
H3_C3_N1_HX	65.67	197.01	0.00	-262.68
H4_C4_N1_HX	65.67	197.01	0.00	-262.68
H1_C1_OH_HO	88.52	265.69	0.00	-354.33
HO_OH_C5_C3	-53.40	461.01	87.56	-495.17
HO_OH_C5_H5	88.52	265.69	0.00	-354.33
N1_C2_C1_H1	96.58	289.86	0.00	-386.44
N1_C2_C1_OH	2012.91	-2012.91	0.00	0.00
OH_C5_C3_H3	117.75	353.24	0.00	-470.99
OH_C5_C3_N1	2012.91	-2012.91	0.00	0.00

S5 Accounting for CO_2 Evaporation in Sequential Absorption of CO_2 (First) and H_2S (Second)

Dicko et al.[36] measured binary absorption of CO_2 and H_2S sequentially. In their measurements, Dicko et al.[36] first loaded 50 wt.% MDEA/water solution with fixed loadings of CO_2 and then loaded H_2S to the CO_2 loaded solution. Dicko et al.[36] assumed that the CO_2 loading in the solution does not change during H_2S absorption. However, CO_2 may evaporate from the solution to the gas phase as the presence of H_2S may shift the equilibrium between CO_2 in the gas phase and free CO_2 in the liquid phase. For multi-component absorption, our chemical reaction equilibrium solver was designed to compute simultaneous absorption of all components in the gas phase. To account for the CO_2 evaporation effect in the experiments by Dicko et al.[36], we modified our solver so it also computes the amount of evaporated CO_2 to the gas phase during loading with H_2S. In these calculations, we first computed the speciation of a CO_2 loaded 50.% MDEA/water solution at 323.15 K for fixed loadings of CO_2 (CO_2 loadings of 0.093, 0.306, 0.510, and 0.706 $\text{mol}_{\text{CO}_2}\ \text{mol}_{\text{amine}}^{-1}$) as explained in the methodology section of the main text. Next, we computed the speciation of the H_2S/CO_2/MDEA/water system using the speciation obtained in the previous computation as an initial guess. In computing the speciation of the H_2S/CO_2/MDEA/water system at equilibrium, we have 11 variables (N_i) and 11 equations to solve. To account for the effect of CO_2 evaporation in the experiments of Dicko et al.[36], we have one more variable additional to the concentrations of the species in liquid phase (N_i), which is the amount of CO_2 evaporated to the gas phase ($N_{\text{CO}_2,\text{gas}}$). As a result of this, the CO_2 balance in the system has an additional term $N_{\text{CO}_2,\text{gas}}$. The CO_2 balance in the H_2S/CO_2/MDEA/water system changes from Eq. 9 of the main text to:

$$N_{\text{CO}_2,\text{total}} - \left(N_{\text{CO}_{2(\text{aq.})}} + N_{\text{HCO}_3^-} + N_{\text{CO}_3^{2-}} + N_{\text{CO}_2,\text{gas}}\right) = 0 \tag{S23}$$

We also have an additional equation derived using the chemical equilibrium between the free CO_2 absorbed in the liquid solution and the CO_2 evaporated to the gas phase and the equilibrium condition (Eq. 1 of the main text):

$$CO_{2(g)} \rightleftharpoons CO_{2(aq.)} \tag{SR1}$$

The chemical equilibrium between the free CO_2 absorbed in the liquid solution and the CO_2 evaporated to the gas phase can be shown as:

$$\frac{RTN_{CO_2,gas}}{V_{gas}} = \frac{N_{CO_{2(aq.)}}RT}{V_{liquid}\exp\left[\frac{-\mu^{ex}_{CO_2}}{RT}\right]} \tag{S24}$$

which results in:

$$\frac{N_{CO_2,liquid}}{N_{CO_2,gas}} = \frac{V_{liquid}}{V_{gas}}\exp\left[\frac{-\mu^{ex}_{CO_2,liquid}}{RT}\right] \tag{S25}$$

where $N_{CO_2,liquid}$ represent the number of molecules of free CO_2 in liquid phase, V_{liquid} is the volume of the liquid phase, V_{gas} is the volume of the gas phase, and $\mu^{ex}_{CO_2,liquid}$ is the excess chemical potential of CO_2 in liquid phase. Note that we used a $\frac{V_{liquid}}{V_{gas}}$ value of 0.3 in these calculations to mimic the conditions in the experiments by Dicko et al.[36]. In total, we have 12 variables and 12 equations to solve in computing the speciation of the H_2S/CO_2/MDEA/water system while accounting for the effect of evaporating CO_2 during sequential H_2S absorption. We solved the speciation in this system using a numerical least squares solver for nonlinear equations. Fig. S5 shows the CO_2 loading in 50 wt.% MDEA/water solution at 323.15 K as a function of H_2S loading during H_2S absorption and comparison with the fixed loading assumption. Results show that the amount of evaporated CO_2 is the highest for the highest initial CO_2 loaded solution. For the solution with initial CO_2 loading of 0.093 $\text{mol}_{CO_2}\,\text{mol}^{-1}_{amine}$, the decrease in CO_2 loading is between 0.6–26.9% of the initial loading ($5.58\times10^{-4} - 2.50\times10^{-2}$ $\text{mol}_{CO_2}\,\text{mol}^{-1}_{amine}$). The decrease in the CO_2 loading is between 9.5–44.6% of the initial CO_2 loading ($6.71\times10^{-2} - 3.14\times10^{-1}$ $\text{mol}_{CO_2}\,\text{mol}^{-1}_{amine}$)

for the solution with the highest initial CO_2 loading (0.706 $mol_{CO_2}\ mol^{-1}_{amine}$) Fig. S6 shows the H_2S partial pressure as a function of the H_2S loading at 323.15 K for the fixed CO_2 loading assumption (no CO_2 evaporation), by quantifying the effect of CO_2 evaporation, and experimental results from Dicko et al.[36]. The computed H_2S partial pressures for fixed CO_2 loading assumptions are always higher than the H_2S partial pressures computed quantifying the effect of CO_2 evaporation. This is expected because there is a lower amount of CO_2 for H_2S to compete when we take the effect of CO_2 evaporation into account, so H_2S can be absorbed by the solution at lower pressures. However, even with the decrease in H_2S partial pressures, the computed H_2S isotherms does not agree with the experimental isotherms[36].

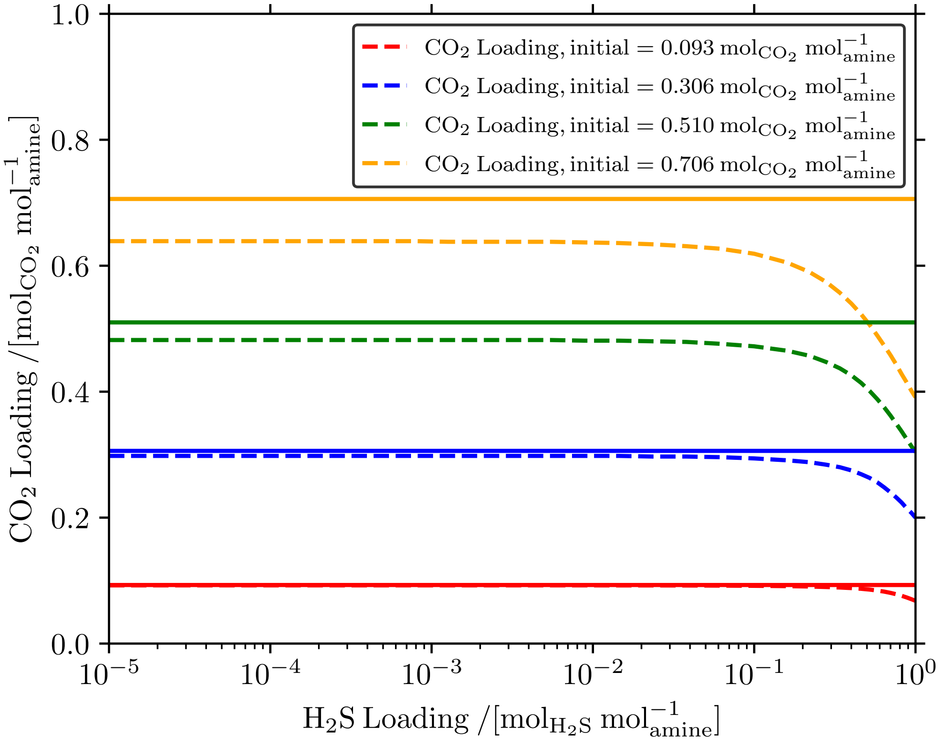

Figure S5: Calculated CO_2 loadings as a function of the H_2S loadings during H_2S absorption in CO_2 loaded 50 wt.% MDEA/water solutions at 323.15 K. The dashed lines show the CO_2 loading after the correction (desorption of CO_2 due to absorption of H_2S) while solid lines show the fixed CO_2 loading assumption.

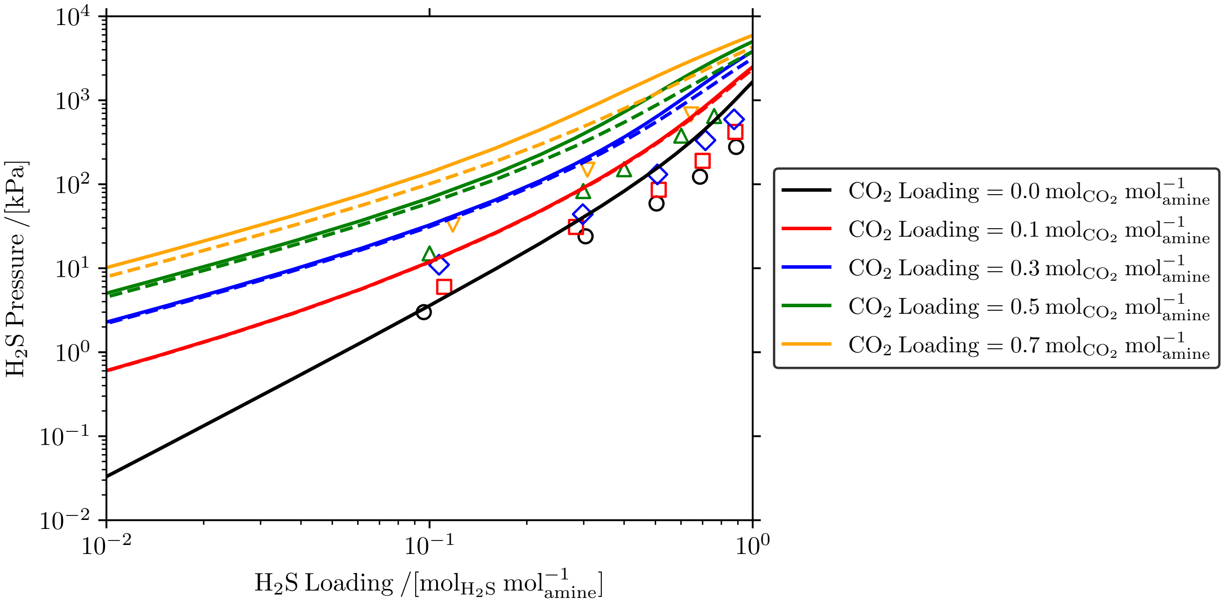

Figure S6: H_2S pressure as a function of H_2S loading. Solid lines represent simultaneous absorption of CO_2 and H_2S while dashed lines represent sequential absorption of CO_2 and then H_2S (accounting for the effect of evaporated CO_2). Empty symbols represent the experimental results from Dicko et al.[36]. Color codings for solid lines, dashed lines, and empty symbols follow the color coding in the legend.

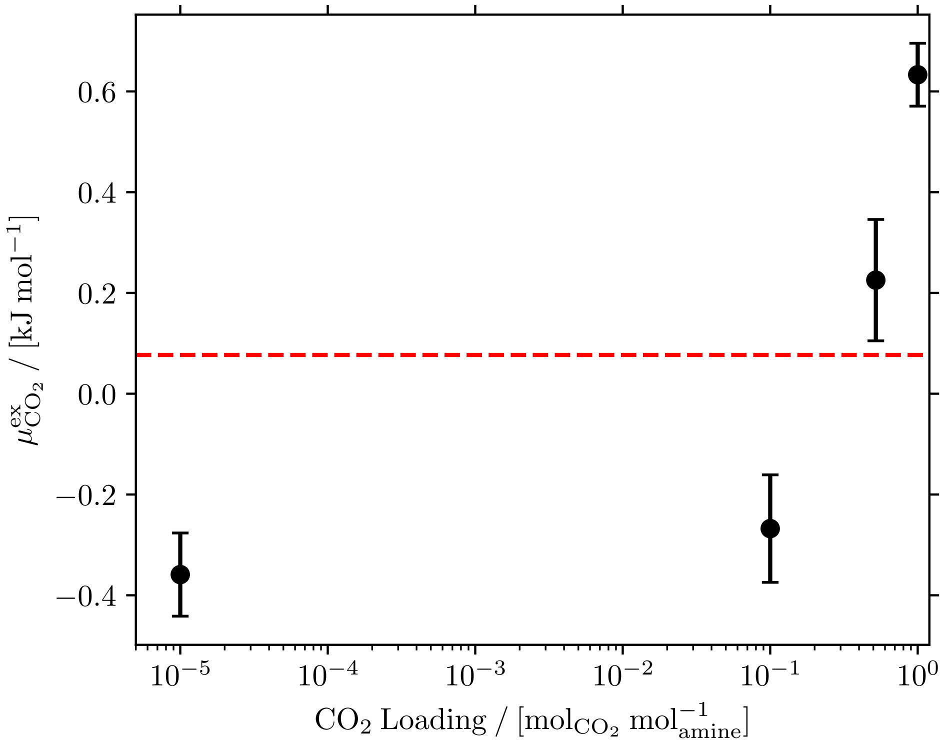

Figure S7: The values of μ_i^{ex} for CO_2 as a function of CO_2 loading in 23 wt.% MDEA/water solutions at 313.15 K. To compute the values of μ_i^{ex} for CO_2 for different CO_2 loadings, we changed the composition of the simulation boxes to the speciations reported in Fig. 3 of the main text. The dashed line represents the computed value of μ_i^{ex} for CO_2 in pure water at 313.15 K.

S6 Derivation of an Expression for the Henry Constant of CO_2 in Aqueous MDEA Solutions

Our aim is to derive an approximate expression for the Henry constant of CO_2 in aqueous MDEA solutions as a function of the equilibrium constants (K) of reactions R1–R4 of the main text. The Henry constant of CO_2 in aqueous MDEA solutions ($K^{\mathrm{H}}_{\mathrm{CO_2}}$) can be expressed as:

$$K^{\mathrm{H}}_{\mathrm{CO_2}} = \lim_{P_{\mathrm{CO_2}} \to 0} \frac{P_{\mathrm{CO_2}}}{X_{\mathrm{CO_2,total}}} \tag{S26}$$

where $P_{\mathrm{CO_2}}$ is the partial pressure of CO_2 in the gas phase and $X_{\mathrm{CO_2,total}}$ is the total mole fraction of the free CO_2, HCO_3^-, and CO_3^{2-} ($X_{\mathrm{CO_2,total}} = X_{\mathrm{CO_2}} + X_{\mathrm{HCO_3^-}} + X_{\mathrm{CO_3^{2-}}}$). There is already an expression to compute $P_{\mathrm{CO_2}}$ (Eq. 7 of the main text), so we need to derive an expression for $X_{\mathrm{CO_2,total}}$. As $P_{\mathrm{CO_2}}$ approaches 0, we assume that the solution is only composed of water and MDEA ($X_{\mathrm{MDEA}} + X_{\mathrm{H_2O}} = 1$) and the net charge of the OH^- ion is the only negative charge in the solution that balances the positive charge from the $MDEAH^+$ ion ($X_{\mathrm{MDEAH^+}} = X_{\mathrm{OH^-}}$) i.e. MDEA is a weak base. By using the equilibrium constant of reaction R4 (K_{R4}) and replacing $X_{\mathrm{OH^-}}$ with $X_{\mathrm{MDEAH^+}}$, we obtain an expression for the mole fraction of the H_3O^+ ion:

$$X_{\mathrm{H_3O^+}} = \frac{K_{\mathrm{R4}} X^2_{\mathrm{H_2O}}}{X_{\mathrm{MDEAH^+}}} \tag{S27}$$

By replacing the term $X_{\mathrm{H_3O^+}}$ with Eq. (S27) in the equilibrium constant of reaction R3 (K_{R3}), an expression to compute the mole fraction of $MDEAH^+$ is obtained:

$$X_{\mathrm{MDEAH^+}} = \sqrt{\frac{K_{\mathrm{R4}} X_{\mathrm{H_2O}} X_{\mathrm{MDEA}}}{K_{\mathrm{R3}}}} \tag{S28}$$

The mole fraction of free CO_2 in the solution can be computed using Eq. 7 of the main text and total number of molecules ($N_{\mathrm{MDEA}} + N_{\mathrm{H_2O}}$) as:

$$X_{\mathrm{CO_2}} = \frac{P_{\mathrm{CO_2}} V \exp\left[\frac{-\mu^{\mathrm{ex}}_{\mathrm{CO_2}}}{RT}\right]}{k_{\mathrm{B}} T (N_{\mathrm{MDEA}} + N_{\mathrm{H_2O}})} \tag{S29}$$

where V is the volume of the liquid phase, $\mu^{\mathrm{ex}}_{\mathrm{CO_2}}$ is the excess chemical potential of CO_2, k_{B} is the Boltzmann constant, and T is the absolute temperature. By using the equilibrium constant of reaction R1 (K_{R1}), we obtain an expression to compute the mole fraction of HCO_3^- which is:

$$X_{\mathrm{HCO_3^-}} = \frac{K_{\mathrm{R1}} X_{\mathrm{CO_2}} X^2_{\mathrm{H_2O}}}{X_{\mathrm{H_3O^+}}} \tag{S30}$$

By using the equilibrium constant of reaction R2 (K_{R2}), we can compute the mole fraction of CO_3^{2-} as:

$$X_{\mathrm{CO_3^{2-}}} = \frac{K_{\mathrm{R2}} X_{\mathrm{HCO_3^-}} X_{\mathrm{H_2O}}}{X_{\mathrm{H_3O^+}}} \tag{S31}$$

By summing Eq. (S29), Eq. (S30), and Eq. (S31) up, an expression for $X_{\mathrm{CO_2,total}}$ is obtained:

$$X_{\mathrm{CO_2,total}} = \frac{P_{\mathrm{CO_2}} V \exp\left[\frac{-\mu^{\mathrm{ex}}_{\mathrm{CO_2}}}{RT}\right]}{k_{\mathrm{B}} T (N_{\mathrm{MDEA}} + N_{\mathrm{H_2O}})} + \frac{K_{\mathrm{R1}} X_{\mathrm{CO_2}} X^2_{\mathrm{H_2O}}}{X_{\mathrm{H_3O^+}}} + \frac{K_{\mathrm{R2}} X_{\mathrm{HCO_3^-}} X_{\mathrm{H_2O}}}{X_{\mathrm{H_3O^+}}} \tag{S32}$$

Because we show the CO_2 pressure as a function of the CO_2 loading in our isotherms (see Fig. 5 of the main text), we prefer to compute the Henry coefficient of CO_2 as:

$$K^{\mathrm{H}}_{\mathrm{CO_2}} = \lim_{P_{\mathrm{CO_2}} \to 0} \frac{P_{\mathrm{CO_2}}}{\alpha_{\mathrm{CO_2,total}}} \tag{S33}$$

where $\alpha_{\mathrm{CO_2,total}}$ is the total loading of CO_2 in the solution in the units of $\mathrm{mol_{CO_2}\ mol^{-1}_{amine}}$ ($\alpha_{\mathrm{CO_2,total}} = \alpha_{\mathrm{CO_2}} + \alpha_{\mathrm{HCO_3^-}} + \alpha_{\mathrm{CO_3^{2-}}}$ where $\alpha_{\mathrm{CO_2}}$, $\alpha_{\mathrm{HCO_3^-}}$, and $\alpha_{\mathrm{CO_3^{2-}}}$ are the loadings of free CO_2, HCO_3^-, and CO_3^{2-}, respectively). The mole fractions computed using Eq. (S29),

Eq. (S30), and Eq. (S31) can be converted to loading α_i using:

$$\alpha_i = X_i \frac{(N_{\mathrm{H_2O}} + N_{\mathrm{MDEA}})}{N_{\mathrm{MDEA}}} \tag{S34}$$

When we evaluate Eq. (S27)–Eq. (S31) using the equilibrium constants of the reactions R1–R4, V, and $\mu^{\mathrm{ex}}_{\mathrm{CO_2}}$ at 313.15 K and 1.2×10^{-7} kPa for 23 wt.% MDEA/water solutions ($X_{\mathrm{H_2O}} = 0.957$ and $X_{\mathrm{MDEA}} = 0.043$), the approximate mole fractions of the species in CO_2/MDEA/water system are computed. Table S29 shows the mole fractions of the species and the Henry constant of CO_2 in CO_2/MDEA/water system computed using Eq. (S27)–Eq. (S31) and our solver at 313.15 K and a low pressure of 1.2×10^{-7} kPa in 23 wt.% MDEA/water solutions. The results show that the mole fractions and Henry constant computed using Eq. (S27)–Eq. (S31) are in excellent agreement with the mole fractions and Henry constant computed numerically using our solver.

Table S29: The mole fractions of species and Henry constants of CO_2 computed using Eq. (S27)–Eq. (S31) and our solver at 313.15 K and 1.2×10^{-7} kPa in 23 wt.% MDEA/water solutions

	from Eq. (S27)–Eq. (S31)	from CASpy
$X_{\mathrm{MDEAH^+}}$	6.25×10^{-5}	6.29×10^{-5}
$X_{\mathrm{H_3O^+}}$	1.62×10^{-13}	1.38×10^{-13}
$X_{\mathrm{OH^-}}$	6.25×10^{-5}	6.21×10^{-5}
$X_{\mathrm{CO_2}}$	7.10×10^{-13}	7.10×10^{-13}
$X_{\mathrm{HCO_3^-}}$	4.35×10^{-8}	5.09×10^{-8}
$X_{\mathrm{CO_3^{2-}}}$	2.78×10^{-7}	3.81×10^{-7}
$K^{\mathrm{H}}_{\mathrm{CO_2}}$ / kPa $\mathrm{mol_{amine}}$ $\mathrm{mol^{-1}_{CO_2}}$	0.0162	0.0149

Using the expression for the Henry constant, the heat of absorption of species i (ΔH_i) can computed using:

$$\Delta H_i = \frac{\mathrm{d}\left(\ln\left[\frac{K^{\mathrm{H}}_i}{K_0}\right]\right)}{\mathrm{d}\left(\frac{1}{RT}\right)} \tag{S35}$$

where K^{H}_i is the Henry constant of species i and K_0 is the arbitrary reference Henry constant to make the argument of the logarithm dimensionless. However, we feel that computing the

heat of absorption is out of the scope of this work.

Table S30: Correlations reported by Plakia et al.[37] to compute mole fraction-based equilibrium constants of reactions R1-R6 of the main text. The mole fraction-based equilibrium constants are computed using the expression $\ln[K_j] = A + \frac{B}{T} + C\ln[T]$ where T is the absolute temperature.

Reaction	A	B	C
R1	231.465	-12092.1	-36.7816
R2	216.049	-12431.7	-35.4819
R3	-83.4914	-819.7	10.9756
R4	132.899	-13445.9	-22.4773
R5	214.582	-12995.4	-33.5471
R6	-32	-3338	0

Table S31: Computed μ_i^{ex} of CO_2 (TraPPE[30] force field with optimized potential for CO_2–H_2O interactions[32])and H_2S (force field from Kristóf and Lizsi[31]) in water (TIP3P[22] force field) as a function of temperature at 1 bar.

	298.15 K	313.15 K	323.15 K
$\mu_{CO_2}^{\text{ex}}$ / [kJ mol^{-1}]	-0.563	0.077	0.410
$\mu_{H_2S}^{\text{ex}}$ / [kJ mol^{-1}]	-2.514	-2.025	-1.786

Table S32: Comparison of the values of μ_i^0 and $D_{0,i}$ computed using quantum chemistry calculations (with the G4 method) and JANAF tables at 313.15 K.

Species	μ_{G4}^0 / [kJ mol^{-1}]	μ_{JANAF}^0 / [kJ mol^{-1}]	$D_{0,i}^{\text{G4}}$ / [kJ mol^{-1}]	$D_{0,i}^{\text{JANAF}}$ / [kJ mol^{-1}]
MDEA	-7758.70	N/A	7625.75	N/A
$MDEAH^+$	-7428.61	N/A	7338.39	N/A
H_2O	-965.23	-986.83	916.06	917.78
H_3O^+	-338.21	-383.14	284.91	312.91
CO_2	-1657.34	-1674.24	1601.81	1597.92
OH^-	-649.47	-662.88	607.62	600.25

Table S33: Comparison of the computed values of μ_i^{ex} in water with available data from literature and μ_i^{ex} values derived from Henry constants at 298.15 K and 1 bar.

Species	Force field	μ_i^{ex} (our calculations) / [kJ mol^{-1}]	μ_i^{ex} (literature[3]) / [kJ mol^{-1}]	μ_i^{ex} (from Henry constants) / [kJ mol^{-1}]
MDEA	GAFF[24]	-43.77	-43.01	N/A
H_2O	TIP3P[22]	-27.83	-28.09	N/A
CO_2	TraPPE[30]	-0.60	1.25	0.44[38]
H_2S	K-L[31]	-2.63	N/A	-2.40[39]

Table S34: Comparison of μ_i^0, $D_{0,i}$, and μ_i^G values computed using different composite methods in quantum chemistry calculations at 313.15 K.

Species	μ^0_{G3} / [kJ mol^{-1}]	μ^0_{G4} / [kJ mol^{-1}]	$\mu^0_{CBS-QB3}$ / [kJ mol^{-1}]	μ^0_{G3B3} / [kJ mol^{-1}]	Standard deviation
MDEA	-7756.55	-7758.7	-7760.17	-7755.58	2.05
H_2O	-964.11	-965.23	-964.48	-968.09	1.80
$MDEAH^+$	-7425.49	-7428.61	-7431.06	-7425.80	2.62
CO_2	-1655.89	-1657.34	-1659.49	-1654.16	2.26
H_3O^+	-339.33	-338.21	-336.76	-340.26	1.51
	D_0^{G3} / [kJ mol^{-1}]	D_0^{G4} / [kJ mol^{-1}]	$D_0^{CBS-QB3}$ / [kJ mol^{-1}]	D_0^{G3B3} / [kJ mol^{-1}]	Standard deviation
MDEA	7653.64	7652.75	7652.14	7652.81	0.62
H_2O	915.87	916.06	917.01	916.30	0.50
$MDEAH^+$	7338.86	7338.39	7338.59	7338.49	0.20
CO_2	1602.40	1601.81	1600.93	1601.58	0.61
H_3O^+	284.64	284.91	285.47	285.45	0.41
Species	μ^G_{G3} / [kJ mol^{-1}]	μ^G_{G4} / [kJ mol^{-1}]	$\mu^G_{CBS-QB3}$ / [kJ mol^{-1}]	μ^G_{G3B3} / [kJ mol^{-1}]	Standard deviation
MDEA	-7784.76	-7786.91	-7788.38	-7783.79	2.05
H_2O	-992.32	-993.44	-992.69	-996.30	1.80
$MDEAH^+$	-7453.70	-7456.82	-7459.27	-7454.01	2.62
CO_2	-1684.10	-1685.55	-1687.70	-1682.37	2.26
H_3O^+	-367.54	-366.42	-364.97	-368.47	1.51

Table S35: Linear regression fit parameters of the values of μ_i^{ex} as a function of charge scaling factor χ. The analytic expression to compute μ_i^{ex} is: $\mu_i^{\mathrm{ex}} = A \times \chi + B$. The values of A and B shown in this table are in $\mathrm{kJ\,mol^{-1}}$.

	GAFF			OPLS-AA		
	A	B	R^2	A	B	R^2
$\mu^{\mathrm{ex}}_{\mathrm{MDEA}}$	-31.40	-13.39	0.963	-68.24	37.51	0.999
$\mu^{\mathrm{ex}}_{\mathrm{MDEAH^{+}+HCO_3^-}}$	-1030.80	444.46	0.997	-988.89	440.99	0.998
$\mu^{\mathrm{ex}}_{\mathrm{H_3O^{+}+HCO_3^-}}$	-1392.01	620.51	0.997	-1392.01	620.51	0.997

Table S36: Linear regression fit parameters of the values of the equilibrium constant of the $\mathrm{MDEAH^+}$ dissociation reaction ($\ln\left[K_{\mathrm{R3,des}}\right]$) as a function of charge scaling factor χ. The analytic expression to compute $\ln\left[K_{\mathrm{R3,des}}\right]$ is: $\ln\left[K_{\mathrm{R3,des}}\right] = A \times \chi + B$.

	A	B	R^2
$\ln\left[K_{\mathrm{R3,des}}\right]$ (GAFF-only the ions are scaled)	139.24	-174.46	0.998
$\ln\left[K_{\mathrm{R3,des}}\right]$ (GAFF-the ions and MDEA are scaled)	175.48	-211.18	0.998
$\ln\left[K_{\mathrm{R3,des}}\right]$ (OPLS-AA-only the ions are scaled)	154.83	-180.62	0.998
$\ln\left[K_{\mathrm{R3,des}}\right]$ (OPLS-AA-the ions and MDEA are scaled)	181.02	-206.97	0.997

References

(1) Smith, W.; Missen, R. *Chemical Reaction Equilibrium Analysis: Theory and Algorithms*, 1st ed.; Wiley: New York, 1982.

(2) Hens, R.; Rahbari, A.; Caro-Ortiz, S.; Dawass, N.; Erdös, M.; Poursaeidesfahani, A.; Salehi, H. S.; Celebi, A. T.; Ramdin, M.; Moultos, O. A.; Dubbeldam, D.; Vlugt, T. J. H. Brick-CFCMC: Open Source Software for Monte Carlo Simulations of Phase and Reaction Equilibria Using the Continuous Fractional Component Method. *Journal of Chemical Information and Modeling* **2020**, *60*, 2678–2682.

(3) Noroozi, J.; Smith, W. R. Accurately Predicting CO_2 Reactive Absorption Properties in Aqueous Alkanolamine Solutions by Molecular Simulation Requiring No Solvent Experimental Data. *Industrial and Engineering Chemistry Research* **2020**, *59*, 18254–18268.

(4) Frenkel, D.; Smit, B. *Understanding molecular simulation: from algorithms to applications*, 2nd ed.; Academic Press: San Diego, California, 2002; Vol. 1.

(5) Polat, H. M.; Salehi, H. S.; Hens, R.; Wasik, D. O.; Rahbari, A.; De Meyer, F.; Houriez, C.; Coquelet, C.; Calero, S.; Dubbeldam, D.; Moultos, O. A.; Vlugt, T. J. H. New Features of the Open Source Monte Carlo Software Brick-CFCMC: Thermodynamic Integration and Hybrid Trial Moves. *Journal of Chemical Information and Modeling* **2021**, *61*, 3752–3757.

(6) Jakobsen, J. P.; Krane, J.; Svendsen, H. F. Liquid-phase composition determination in CO_2-H_2O-alkanolamine systems: An NMR study. *Industrial and Engineering Chemistry Research* **2005**, *44*, 9894–9903.

(7) Chase, M. W.; Curnutt, J. L.; Prophet, H.; Mcdonald, R. A.; Syverud, A. N. JANAF thermochemical tables, 1975 supplement. *Journal of Physical and Chemical Reference Data* **1975**, *4*, 1–176.

(8) Chase, M. *NIST-JANAF Thermochemical Tables*, 4th ed.; American Institute of Physics: New York, 1998.

(9) McQuarrie, D. A.; Simon, J. D. *Physical chemistry: a molecular approach*; University Science Books: Sausalito, California, 1997.

(10) Frisch, M. J.; Trucks, G. W.; Schlegel, H. B.; Scuseria, G. E.; Robb, M. A.; Cheeseman, J. R.; Scalmani, G.; Barone, V.; Mennucci, B.; Petersson, G. A.; Nakatsuji, H.; Caricato, M.; Li, X.; Hratchian, H. P.; Izmaylov, A. F.; Bloino, J.; Zheng, G.; Sonnenberg, J. L.; Hada, M.; Ehara, M.; Toyota, K.; Fukuda, R.; Hasegawa, J.; Ishida, M.; Nakajima, T.; Honda, Y.; Kitao, O.; Nakai, H.; Vreven, T.; Montgomery, J. A., Jr.; Peralta, J. E.; Ogliaro, F.; Bearpark, M.; Heyd, J. J.; Brothers, E.; Kudin, K. N.; Staroverov, V. N.; Kobayashi, R.; Normand, J.; Raghavachari, K.; Rendell, A.; Burant, J. C.; Iyengar, S. S.; Tomasi, J.; Cossi, M.; Rega, N.; Millam, J. M.; Klene, M.; Knox, J. E.; Cross, J. B.; Bakken, V.; Adamo, C.; Jaramillo, J.; Gomperts, R.; Stratmann, R. E.; Yazyev, O.; Austin, A. J.; Cammi, R.; Pomelli, C.; Ochterski, J. W.; Martin, R. L.; Morokuma, K.; Zakrzewski, V. G.; Voth, G. A.; Salvador, P.; Dannenberg, J. J.; Dapprich, S.; Daniels, A. D.; Farkas, Ö.; Foresman, J. B.; Ortiz, J. V.; Cioslowski, J.; Fox, D. J. Gaussian 09 Revision E.01. Gaussian Inc. Wallingford CT 2009.

(11) Perdew, J. P.; Ernzerhof, M.; Zupan, A.; Burke, K. Why Density-Gradient Corrections Improve Atomization Energies and Barrier Heights. *Advances in Quantum Chemistry* **1998**, *33*, 1–9.

(12) Rahbari, A.; Hens, R.; Ramdin, M.; Moultos, O. A.; Dubbeldam, D.; Vlugt, T. J. H. Recent advances in the Continuous Fractional Component Monte Carlo methodology. *Molecular Simulation* **2021**, *47*, 804–823.

(13) Smith, W. R.; Missen, R. W. The effect of isomerization on chemical equilibrium. *The Canadian Journal of Chemical Engineering* **1974**, *52*, 280–282.

(14) Peterson, K. A.; Feller, D.; Dixon, D. A. Chemical accuracy in ab initio thermochemistry and spectroscopy: current strategies and future challenges. *Theoretical Chemistry Accounts* **2012**, *131*, 1–20.

(15) Shi, W.; Maginn, E. J. Continuous Fractional Component Monte Carlo: An adaptive biasing method for open system atomistic simulations. *Journal of Chemical Theory and Computation* **2007**, *3*, 1451–1463.

(16) Shi, W.; Maginn, E. J. Improvement in molecule exchange efficiency in Gibbs Ensemble Monte Carlo: Development and implementation of the Continuous Fractional Component move. *Journal of Computational Chemistry* **2008**, *29*, 2520–2530.

(17) Rosch, T. W.; Maginn, E. J. Reaction Ensemble Monte Carlo simulation of complex molecular systems. *Journal of Chemical Theory and Computation* **2011**, *7*, 269–279.

(18) Allen, M. P.; Tildesley, D. J. *Computer Simulation of Liquids*, 2nd ed.; Clarendon Press: New York, NY, USA, 2017.

(19) Ewald, P. P. Die Berechnung optischer und elektrostatischer Gitterpotentiale. *Annalen der Physik* **1921**, *369*, 253–287.

(20) Siepmann, J. I.; Mcdonald, I. R.; Frenkel, D. Finite-size corrections to the chemical potential. *Journal of Physics: Condensed Matter* **1992**, *4*, 679–691.

(21) Young, J. M.; Panagiotopoulos, A. Z. System-Size Dependence of Electrolyte Activity Coefficients in Molecular Simulations. *Journal of Physical Chemistry B* **2018**, *122*, 3330–3338.

(22) Jorgensen, W. L.; Chandrasekhar, J.; Madura, J. D.; Impey, R. W.; Klein, M. L. Comparison of simple potential functions for simulating liquid water. *Journal of Chemical Physics* **1983**, *79*, 926.

(23) Rahbari, A.; Poursaeidesfahani, A.; Torres-Knoop, A.; Dubbeldam, D.; Vlugt, T. J. H. Chemical potentials of water, methanol, carbon dioxide and hydrogen sulphide at low temperatures using continuous fractional component Gibbs Ensemble Monte Carlo. *Molecular Simulation* **2018**, *44*, 405–414.

(24) Wang, J.; Wolf, R. M.; Caldwell, J. W.; Kollman, P. A.; Case, D. A. Development and testing of a general amber force field. *Journal of Computational Chemistry* **2004**, *25*, 1157–1174.

(25) Jorgensen, W. L.; Maxwell, D. S.; Tirado-Rives, J. Development and testing of the OPLS all-atom force field on conformational energetics and properties of organic liquids. *Journal of the American Chemical Society* **1996**, *118*, 11225–11236.

(26) Rizzo, R. C.; Jorgensen, W. L. OPLS all-atom model for amines: Resolution of the amine hydration problem. *Journal of the American Chemical Society* **1999**, *121*, 4827–4836.

(27) Besler, B. H.; Merz, K. M.; Kollman, P. A. Atomic charges derived from semiempirical methods. *Journal of Computational Chemistry* **1990**, *11*, 431–439.

(28) Wang, J.; Wang, W.; Kollman, P. A.; Case, D. A. Automatic atom type and bond type perception in molecular mechanical calculations. *Journal of Molecular Graphics and Modelling* **2006**, 247–260.

(29) Dodda, L. S.; Vaca, I. C. D.; Tirado-Rives, J.; Jorgensen, W. L. LigParGen web server: an automatic OPLS-AA parameter generator for organic ligands. *Nucleic Acids Research* **2017**, *45*, W331–W336.

(30) Potoff, J. J.; Siepmann, J. I. Vapor–liquid equilibria of mixtures containing alkanes, carbon dioxide, and nitrogen. *AIChE Journal* **2001**, *47*, 1676–1682.

(31) Kristóf, T.; Liszi, J. Effective Intermolecular Potential for Fluid Hydrogen Sulfide. *Journal of Physical Chemistry B* **1997**, *101*, 5480–5483.

(32) Orozco, G. A.; Economou, I. G.; Panagiotopoulos, A. Z. Optimization of intermolecular potential parameters for the CO_2/H_2O mixture. *Journal of Physical Chemistry B* **2014**, *118*, 11504–11511.

(33) Ryckaert, J. P.; Bellemans, A. Molecular dynamics of liquid n-butane near its boiling point. *Chemical Physics Letters* **1975**, *30*, 123–125.

(34) Noroozi, J.; Smith, W. R. Force Field-Based Computational Study of the Thermodynamics of a Large Set of Aqueous Alkanolamine Solvents for Post-Combustion CO_2 Capture. *Journal of Chemical Information and Modeling* **2021**, *61*, 4497–4513.

(35) Dodda, L. S.; Vilseck, J. Z.; Tirado-Rives, J.; Jorgensen, W. L. 1.14*CM1A-LBCC: Localized Bond-Charge Corrected CM1A Charges for Condensed-Phase Simulations. *Journal of Physical Chemistry B* **2017**, *121*, 3864–3870.

(36) Dicko, M.; Coquelet, C.; Jarne, C.; Northrop, S.; Richon, D. Acid gases partial pressures above a 50wt% aqueous methyldiethanolamine solution: Experimental work and modeling. *Fluid Phase Equilibria* **2010**, *289*, 99–109.

(37) Plakia, A.; Voutsas, E. Modeling of H_2S, $CO_2 + H_2S$, and $CH_4 + CO_2$ Solubilities in Aqueous Monoethanolamine and Methyldiethanolamine Solutions. *Industrial and Engineering Chemistry Research* **2020**, *59*, 11317–11328.

(38) Enick, R. M.; Klara, S. M. CO_2 solubility in water and brine under reservoir conditions. *Chemical Engineering Communications* **1990**, *90*, 23–33.

(39) Clarke, E. C. W.; Glew, D. N. Aqueous Nonelectrolyte Solutions. Part VIII. Deuterium and Hydrogen Sulfides Solubilities in Deuterium Oxide and Water. *Canadian Journal of Chemistry* **1971**, *49*, 691.
